# Cellular Effects of 2′,3′-Cyclic Nucleotide Monophosphates in Gram-Negative Bacteria

**DOI:** 10.1128/JB.00208-21

**Published:** 2022-01-18

**Authors:** Yashasvika Duggal, Jennifer E. Kurasz, Benjamin M. Fontaine, Nick J. Marotta, Shikha S. Chauhan, Anna C. Karls, Emily E. Weinert

**Affiliations:** a Department of Chemistry, Pennsylvania State University, University Park, Pennsylvania, USA; b Department of Microbiology, University of Georgia, Athens, Georgia, USA; c Department of Chemistry, Emory University, Atlanta, Georgia, USA; d Molecular, Cellular and Integrative Biosciences Program, Pennsylvania State University, University Park, Pennsylvania, USA; e Department of Biochemistry and Molecular Biology, Pennsylvania State University, University Park, Pennsylvania, USA; Geisel School of Medicine at Dartmouth

**Keywords:** RNA degradation, RNA repair, biofilms, cyclic nucleotides, flagellar motility, metabolomics, transcriptomics

## Abstract

Organismal adaptations to environmental stimuli are governed by intracellular signaling molecules such as nucleotide second messengers. Recent studies have identified functional roles for the noncanonical 2′,3′-cyclic nucleotide monophosphates (2′,3′-cNMPs) in both eukaryotes and prokaryotes. In Escherichia coli, 2′,3′-cNMPs are produced by RNase I-catalyzed RNA degradation, and these cyclic nucleotides modulate biofilm formation through unknown mechanisms. The present work dissects cellular processes in E. coli and Salmonella enterica serovar Typhimurium that are modulated by 2′,3′-cNMPs through the development of cell-permeable 2′,3′-cNMP analogs and a 2′,3′-cyclic nucleotide phosphodiesterase. Utilization of these chemical and enzymatic tools, in conjunction with phenotypic and transcriptomic investigations, identified pathways regulated by 2′,3′-cNMPs, including flagellar motility and biofilm formation, and by oligoribonucleotides with 3′-terminal 2′,3′-cyclic phosphates, including responses to cellular stress. Furthermore, interrogation of metabolomic and organismal databases has identified 2′,3′-cNMPs in numerous organisms and homologs of the E. coli metabolic proteins that are involved in key eukaryotic pathways. Thus, the present work provides key insights into the roles of these understudied facets of nucleotide metabolism and signaling in prokaryotic physiology and suggest broad roles for 2′,3′-cNMPs among bacteria and eukaryotes.

**IMPORTANCE** Bacteria adapt to environmental challenges by producing intracellular signaling molecules that control downstream pathways and alter cellular processes for survival. Nucleotide second messengers serve to transduce extracellular signals and regulate a wide array of intracellular pathways. Recently, 2′,3′-cyclic nucleotide monophosphates (2′,3′-cNMPs) were identified as contributing to the regulation of cellular pathways in eukaryotes and prokaryotes. In this study, we define previously unknown cell processes that are affected by fluctuating 2′,3′-cNMP levels or RNA oligomers with 2′,3′-cyclic phosphate termini in E. coli and Salmonella Typhimurium, providing a framework for studying novel signaling networks in prokaryotes. Furthermore, we utilize metabolomics databases to identify additional prokaryotic and eukaryotic species that generate 2′,3′-cNMPs as a resource for future studies.

## INTRODUCTION

In all domains of life, organisms respond to stimuli using second messenger signaling pathways in which pools of specialized nucleotides regulate diverse biological functions. For example, guanosine 3′,5′-cyclic monophosphate (3′,5′-cGMP) regulates processes such as vasodilation and visual transduction in eukaryotes (1, 2), while adenosine 3′,5′-cyclic monophosphate (3′,5′-cAMP) governs numerous processes in diverse organisms, including steroidogenesis in mammals and carbon catabolism in bacteria (3, 4). Cyclic dimeric-3′:5′-GMP (c-di-GMP) modulates biofilm formation in many bacteria, and other cyclic dimeric nucleotides control processes such as sporulation in prokaryotes and innate immunity in eukaryotes (5–8). Studies also have demonstrated that acyclic nucleotides in bacteria, including guanosine 3′-diphosphate, 5′-(tri)diphosphate ([p]ppGpp), and P1,P4-di-adenosine 5′-tetraphosphate (Ap4A), induce transcriptome and proteome remodeling (7) and regulate biofilm production via modulation of c-di-GMP levels (9, 10), respectively. In addition to the nucleotide second messengers, primary nucleotide metabolism influences a host of physiological processes in bacteria. Perturbation of *de novo* nucleotide biosynthesis alters biofilm formation in Escherichia coli (11–13), exogenous addition of nucleosides and dNTPs promotes a positive chemotactic response in Vibrio fischeri (14), and pyrimidine nucleobases function as chemoattractants in E. coli (15). These findings illustrate the multifaceted nature of nucleotide signal transduction and suggest that other aspects of nucleotide synthesis and salvage regulate cellular processes, warranting further investigation into the biological effects of additional nucleotide pools.

An intriguing class of nucleotides is the 2′,3′-cyclic nucleotide monophosphates (2′,3′-cNMPs). These were first observed in E. coli several decades ago (16), but exploration of their biological relevance has been limited. Recent studies have demonstrated that 2′,3′-cAMP mediates stress granule assembly in Arabidopsis thaliana (17). Earlier studies in mammals correlated the appearance of extracellular 2′,3′-cAMP with organ stress (18) and revealed that increased intracellular 2′,3′-cAMP levels stimulate Ca^2+^ efflux in rat liver cells and oligodendrocytes, resulting in depolarization of the mitochondrial membrane and concomitant apoptosis (19). In addition, our quantification of 2′,3′-cNMPs in rat organs showed organ-specific variable basal levels for 2′,3′-cAMP, -cGMP, -cCMP, and -cIMP (20). Together, these studies support a role for 2′,3′-cNMPs in regulating eukaryotic physiology; however, less work has been done to characterize the biological significance of these molecules in prokaryotes.

2′,3′-cNMPs have been detected in different bacterial species (2′,3′-cCMP and -cUMP in Pseudomonas fluorescens [21]; 2′,3′-cAMP in Staphylococcus aureus [22]; 2′,3′-cAMP, -cGMP, -cUMP, and -cCMP in E. coli [23]), with our group reporting the physiological quantification of these cyclic nucleotides and mechanism of cellular biosynthesis in E. coli (24). Within E. coli, RNase I, an RNase T2 family member (25), generates all detectable 2′,3′-cNMPs through hydrolysis of RNA, providing the first insight into the biosynthetic origin of these atypical nucleotides in any organism (24). Additional experiments identified physiological functions for 2′,3′-cNMPs and RNase I in biofilm production (24); separately, RNase I was also found to modulate E. coli motility, resistance to β-lactams and acid stress, and outer membrane structure (26). These preliminary findings suggest that there are additional 2′,3′-cNMP- and RNase I-dependent processes yet to be discovered.

In the present study, we investigate the effects of depleting intracellular 2′,3′-cNMP levels in two bacterial species, E. coli and Salmonella enterica serovar Typhimurium (*S.* Typhimurium), using transcriptome sequencing (RNA-seq) to inform a series of physiological assays. We analyzed the transcriptome of a wild-type (WT) strain expressing a heterologous mammalian 2′,3′-cyclic nucleotide phosphodiesterase (CNPase) that exhibits decreased 2′,3′-cNMP concentrations and compared it to an equivalent strain expressing a catalytically inactive CNPase variant (27) (CNPase H73L/H152L, termed “CNP-inact”) that exhibits WT 2′,3′-cNMP levels. CNPase was chosen for its substrate specificity; unlike other cyclic nucleotide phosphodiesterases, it does not hydrolyze 3′,5′-cNMPs and acts primarily on 2′,3′ cyclic phosphates of 2′,3′-cyclic mononucleotides, as well as 2′,3′-cyclic phosphates on the 3′ terminus of oligoribonucleotides (reviewed in reference 28). Both RNase I deletion and expression of CNPase reduce 2′,3′-cNMP levels; however, relative to our recently reported data comparing gene expression in an E. coli Δ*rna* mutant to that in the WT strain (26), a comparison of a WT strain expressing CNPase versus one expressing CNP-inact allows us to uncover processes that are modulated directly by 2′,3′-cyclic phosphate-containing molecules, while excluding any confounding effects of deleting RNase I. This is particularly important, as our previous work identified roles for RNase I that are not linked to cytoplasmic 2′,3′-cNMP levels. Our transcriptomic analysis revealed a large number of genes with diverse cellular functions that are differentially regulated, suggesting that 2′,3′-cNMPs and oligoribonucleotides with 3′-terminal 2′,3′-cyclic phosphates affect a variety of physiological activities. Comparison of CNPase-dependent transcriptome changes in E. coli WT versus Δ*rna* strains demonstrated that most CNPase-dependent differential gene expression is likely to be due to altered levels of 2′,3′-cyclic mononucleotides versus those of oligoribonucleotides with 3′-terminal 2′,3′-cyclic phosphates. These effects were further characterized in subsequent physiological assays, using the expression of CNPase to explore how levels of 2′,3′-cyclic phosphate-containing molecules affect cellular phenotypes. As an additional tool, cell-permeable 2′,3′-cNMP analogs, inspired by lipophilic derivatives of 3′,5′-cAMP and -GMP that are widely employed to study second messenger signaling in eukaryotes (3), were synthesized to further examine 2′,3′-cNMP-dependent phenotypes. Our studies link 2′,3′-cNMPs to the regulation of genes involved in several cellular processes in E. coli, including biofilm formation and motility, highlighting key pathways that are influenced by 2′,3′-cNMP pools and suggesting the presence of 2′,3′-cNMP-sensing proteins within the cell. In addition, our results support regulatory roles for both 2′,3′-cNMPs and RNA oligomers with 2′,3′-cyclic phosphate termini in E. coli responses to stress, including acid tolerance.

Finally, a major point of interest was whether 2′,3′-cNMPs have a common origin or play universal regulatory roles in diverse bacteria. In this study, we examine the topic by exploring similarities and differences in 2′,3′-cNMP generation and physiological impacts in E. coli and *S.* Typhimurium, a closely related but physiologically distinct enteric pathogen. As in E. coli, RNase I plays an essential role in the generation of 2′,3′-cNMPs in *S.* Typhimurium, suggesting a common biosynthetic mechanism in the two species. Analysis of transcriptome-wide changes in WT and Δ*rna S.* Typhimurium strains expressing CNPase or CNP-inact identified common and distinct cellular processes linked to 2′,3′-cyclic phosphate-containing molecules, compared to E. coli, some of which were further addressed with physiological assays. Investigation of existing metabolomic databases for 2′,3′-cNMPs has yielded further insights into their prevalence in other bacteria and eukaryotes, suggesting potential widespread importance of 2′,3′-cNMPs.

## RESULTS

### Transcriptomics reveals 2′,3′-cNMP-dependent processes in E. coli.

Our group previously found that RNase I (encoded by the *rna* gene) generates all detectable 2′,3′-cNMPs in E. coli (26); however, since RNase I affects nucleotide metabolism in both the cytoplasm and the periplasm (24, 26), we required methods to modulate 2′,3′-cNMP levels independently of RNase I expression to determine which cellular effects are directly attributable to fluctuations in 2′,3′-cNMP pools. To this end, we leveraged the catalytic domain of Rattus norvegicus cyclic nucleotide phosphodiesterase (29) (CNPase; UniProtKB P13233 for full-length protein) to hydrolyze 2′,3′-cNMPs in RNase I^+^ (wild-type [WT]) cells. Previous work validated that E. coli expressing CNPase from plasmid pKT-CNP exhibits subquantifiable levels of 2′,3′-cAMP and -cGMP, and ∼25-fold and ∼15-fold lower levels of 2′,3′-cCMP and -cUMP, respectively (24). Therefore, expression of CNPase enabled identification of 2′,3′-cNMP-linked processes through transcriptomic profiling of WT E. coli with reduced 2′,3′-cNMP levels, eliminating the confounding effects inherent to using the E. coli Δ*rna* strain as in our previous transcriptomic analysis (26). RNA-seq revealed 519 genes that were significantly differentially expressed (adjusted *P* [*P*_adj_] < 0.05; >2-fold change in expression) in WT E. coli expressing CNPase compared to expression in equivalent cultures expressing a catalytically inactive CNPase variant (CNP-inact) (see Data Set S1 in the supplemental material). Further transcriptome analysis to address the effects of CNPase hydrolysis of substrates other than 2′,3′-cNMPs is presented below; this analysis supports that CNPase-mediated reduction of 2′,3′-cNMP levels affect gene expression as specified in the following results.

Approximately 13% (*n *= 67) of these identified genes were downregulated in exponential-phase samples expressing active CNPase, which exhibit decreased 2′,3′-cNMP levels (Data Set S1). Of these, only 10 genes were downregulated in both the E. coli WT CNPase strain and the previously analyzed Δ*rna* strain (Data Set S1) (26), suggesting that RNase I contributes to cellular processes in ways that are independent from its role in generating 2′,3′-cNMPs (26). The genes that were most downregulated by CNPase were *napH* (9.08-fold) and *napG* (7.50-fold); together, their gene products produce the NapGH ubiquinol reductase that participates in the Nap periplasmic nitrate reduction pathway during anaerobic growth (Table 1). Expression of *napD*, which encodes a chaperone for NapA, the nitrate reductase catalytic subunit, was additionally downregulated (6.83-fold), suggesting an additional posttranslational level of control that may contribute to regulation of the nitrate reduction pathway (Table 1). Tryptophan biosynthesis was among the other pathways that were substantially downregulated, with RNA-seq identifying decreases in the transcript levels of *trpB* (7.12-fold), *trpA* (6.84-fold), *trpC* (5.41-fold), and *trpD* (4.91-fold) (Table 1). The *trp* operon is well known for its regulation, facilitated by both the Trp repressor and attenuation dependent on intracellular tryptophan levels; strikingly, these data indicate the possibility that 2′,3′-cNMPs additionally affect regulation of this biosynthetic pathway. The mechanisms by which 2′,3′-cNMPs regulate these and other metabolic pathways are a subject of ongoing investigation.

**TABLE 1:** CNPase-dependent differentially expressed genes in E. coli and/or *S.* Typhimurium relevant to this study

Function or gene	Product	log_2_FC WT CNP/CNP-inact (log_2_FC Δ*rna* CNP/CNP-inact) for^*a*^:	
		E. coli	Salmonella Typhimurium
Flagellar assembly			
*fliE*	Flagellar basal body component	1.80	NS
*fliR*	Flagellar export pore protein	1.26	NS
*flgB*	Flagellar basal body rod protein	NS	2.35 (1.17)
*flgC*	Flagellar basal body rod protein	NS	2.33 (1.15)
*flgD*	Flagellar basal body rod modification protein	NS	2.14 (1.18)
*flgJ*	Peptidoglycan hydrolase	NS	1.60
EPS production and/or biofilm			
*csgC*	Curli assembly protein	1.90	NS
*hofC*	Type IV pilin biogenesis assembly protein	1.05	NS
*pgaB*	Poly-beta-1,6-*N*-acetyl-d-glycosamine (PGA) OM export lipoprotein	1.39	NS
*pgaC*	PGA synthase catalytic subunit	1.49	NS
*pgaD*	PGA synthase catalytic subunit	1.22	NS
*sfmA*	Fimbria-like protein	1.77	NS
*sfmH*	Fimbria-like protein	2.43	NS
*yadC*	Putative fimbria-like adhesin protein	1.92	NS
*yadK*	Putative fimbria-like adhesin protein	2.01	NS
*yadL*	Putative fimbria-like adhesin protein	1.48	NS
*yadM*	Putative fimbria-like adhesin protein	1.86	NS
*yadN*	Putative fimbria-like adhesin protein	2.19	NS
*cpsG*	Phosphomannomutase, colanic acid synthesis	NS	−1.81
*gmd*	GDP-d-mannose dehydratase, colanic acid synthesis	NS	−2.03
*manC*	Mannose-1-phosphate guanylyl-transferase, colanic acid synthesis	NS	−1.72
*wcaD*	Putative colanic acid polymerase	1.48	NS
*wcaE*	Glycosyl transferase, colanic acid synthesis	NS	−1.47
*wcaF*	Putative colanic acid biosynthesis acetyltransferase	NS	−1.48
* wcaG*	GDP-fucose synthetase, colanic acid synthesis	NS	−2.06
* wcaI*	Glycosyl transferase, colanic acid synthesis	NS	−1.92
* wzb*	Tyrosine phosphatase, regulator of colanic acid synthesis	NS	−2.13
* yjbE*	Putative EPS production OM protein	1.39	−1.57
* yjbF*	Putative EPS production OM protein	1.37	−1.27
* yjbH*	DUF940 family EPS protein	1.18	NS
Acid resistance			
* cadA*	Lysine decarboxylase	1.74	NS
* cadC*	*cadBA* operon transcriptional activator	1.71 (1.60)	
* aidC*	Arginine/agmatine antiporter	1.42 (3.15)	
* gadA*	Glutamate decarboxylase alpha	1.97 (4.71)	NS
* gadB*	Glutamate decarboxylase beta	2.90 (4.11)	NS
* gadC*	Glutamate/gamma-aminobutyric acid antiporter	2.12 (3.96)	NS
* gadE*	Transcriptional activator of *gad* regulon	2.18	NS
* ybaT*	Glutamine permease	1.20 (2.17)	
* hdeA*	Acid stress response chaperone	2.26 (4.40)	NS
* hdeB*	Acid stress response chaperone	2.58 (4.76)	NS
* hdeD*	Acid resistance IM protein	2.10 (4.39)	NS
* ycaM*	Glutamate/gamma-aminobutyric acid antiporter	1.28	NS
* yceO*	Uncharacterized protein involved in acid stress response	1.95	NS
* yfdE*	Acetyl coenzyme A:oxalate coenzyme A transferase	1.73	NS
* yfdX*	Uncharacterized acid stress protein	1.34	NS
* yhiD*	Putative Mg^2+^ transporter	2.56 (3.76)	NS
* yhiM*	Acid resistance IM protein	1.70 (2.26)	NS
* yhiF (dctR)*	Transcriptional regulator of acid metabolite gene C4 transporter *dctA*	2.36 (3.14)	
* slp*	Acid resistance OM lipoprotein	1.97 (3.31)	
* yjaA*	Acid stress-induced protein	1.50	NS
RNA repair			
* STM14_4239*	Rsr ribonucleoprotein-related protein	NS	−3.67 (−5.70)
* rtcA*	RNA 3′-terminal phosphate cyclase	NS	−3.86 (−4.44)
* rtcB*	RNA ligase	NS	−3.80 (−4.78)
Heat shock response			
* dnaJ*	Chaperone Hsp40	−1.11	−1.63 (−2.36)
* dnaK*	Chaperone Hsp70	−1.34	NS
* ybbN*	DnaK cochaperone	−1.04	NS
* ibpB*	Heat shock chaperone	−1.38	−2.99 (−3.58)
* ibpA*	Heat shock protein	NS	−2.98 (−3.25)
Peroxide stress (SOS response)			
* dinF*	SOS response protein	NS	−1.54
* dinI*	DNA damage-inducible protein	NS	−1.74
* recN*	Recombination and repair protein	NS	−2.21 (−1.59)
* umuC*	DNA polymerase V subunit	NS	−1.30
* umuD*	DNA polymerase V subunit	NS	−1.71
* yebG*	DNA damage-inducible protein	NS	−2.08 (−1.61)
Anaerobic respiration			
* napD*	Assembly protein for nitrate reductase	−2.90	NS
* napG*	Periplasmic nitrate reductase	−2.90	NS
* napH*	Periplasmic nitrate reductase	−3.18	NS
* nirB*	Nitrite reductase subunit	−2.02	NS
Amino acid biosynthesis			
* trpA*	Tryptophan synthase alpha subunit	−2.77	NS
* trpB*	Tryptophan synthase beta subunit	−2.83	NS
* trpC*	Tryptophan biosynthesis protein	−2.44	NS
* trpD*	Tryptophan biosynthesis protein	−2.29	NS
* glnA*	Glutamine synthetase	−1.50	NS

a log_2_ fold changes (log_2_FC) in transcript levels for E. coli or *S.* Typhimurium WT CNP/CNP-inact and Δ*rna* CNP/CNP-inact (in parentheses). NS, indicates that transcript levels changed <2-fold or were not statistically significant.

Approximately 87% (*n *= 452) of the differentially expressed genes were upregulated by the expression of CNPase, with 9 genes that were similarly upregulated in the Δ*rna* mutant (Data Set S1) (26). Several of the most highly upregulated genes are involved in acid resistance, discussed in greater detail below. Others are putatively involved in attachment, such as *sfmA* (5.40-fold) and *sfmH* (3.17-fold), which encode FimA (type 1 fimbrial protein) homologs, and several genes that are predicted to encode fimbria-like adhesin proteins (*n *= 15; *yadN* had the highest fold change at 4.56-fold) or are prophage genes of unknown or unconfirmed function (Table 1; see also Data Set S1).

**FIG 1:**
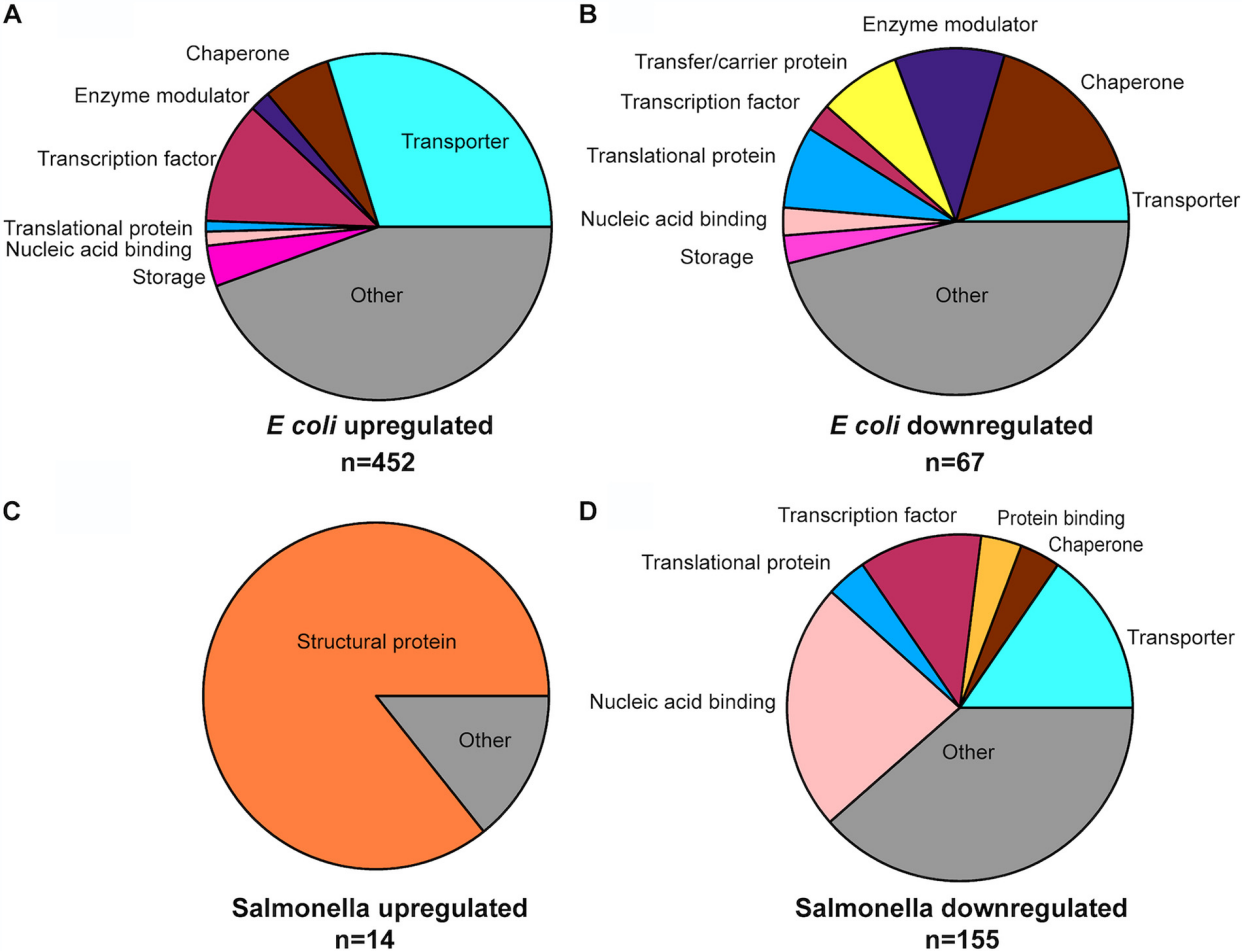
Global gene analysis reveals 2′,3′-cNMP-modulated cellular processes. Functional categorization of differentially expressed genes in Escherichia coli (A and B) and Salmonella Typhimurium (C and D) expressing CNPase compared to those in strains expressing CNP-inact. Pie charts were generated using the Panther Gene Ontology Database (http://pantherdb.org/). Differentially regulated protein classes were determined based on genes that are upregulated by expression of CNPase (A and C) and genes that are downregulated by expression of CNPase (B and D). Gene expression data were obtained from analysis of 3 biological replicates that were significantly differentially expressed (adjusted *P* [*P*_adj_] < 0.05; >2-fold change in expression).

Gene ontology (GO) analysis revealed that 2′,3′-cNMPs modulate genes encoding diverse protein classes; frequent GO terms for primary targets included transcription factor, transporter, hydrolase, transferase, lyase, and oxido-reductase (Fig. 1A). 2′,3′-cNMPs also could be linked to the regulation of genes that impact numerous cell functions, including biofilm formation, motility, and multiple stress responses. These results informed a series of physiological assays to explore phenotypic changes as a response to altering the cyclic nucleotide pools.

### 2′,3′-cNMPs influence flagellar motility in E. coli.

Expression of CNPase led to a slight increase in the mRNA levels of the flagellar-associated genes *fliR* (2.39-fold), which encodes a flagellar export protein, and *fliE* (3.48-fold), which encodes part of the flagellar basal body (Table 1). In contrast to the Δ*rna* strain, which exhibited significant upregulation of genes related to flagellum biosynthesis and chemotaxis (24, 26), decreasing the 2′,3′-cNMP concentration through expression of CNPase did not alter abundance of other flagellum- or chemotaxis-related transcripts.

**FIG 2:**
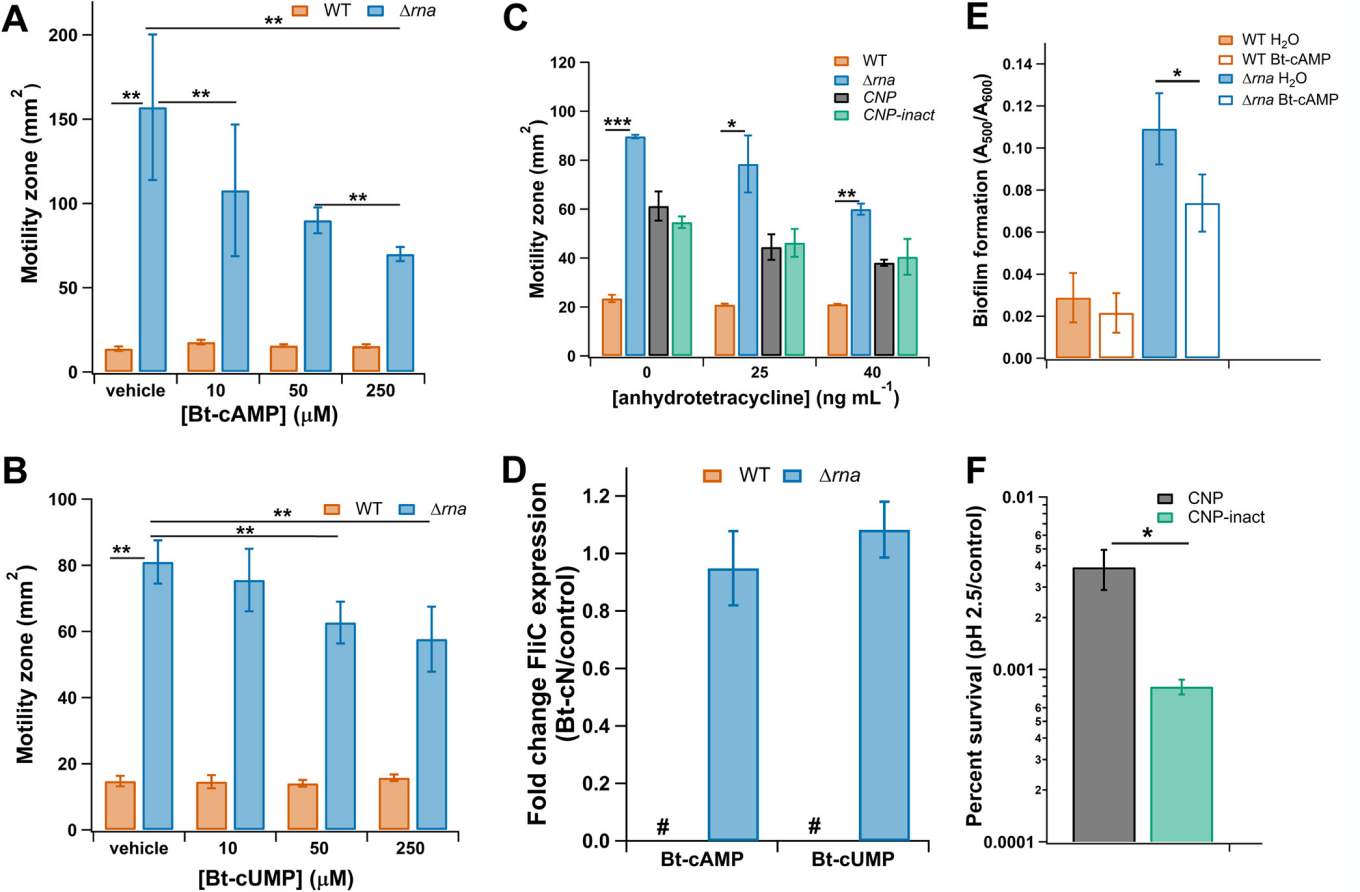
Modulation of 2′,3′-cNMPs impacts E. coli physiology. (A and B) E. coli BW25113 Δ*rna* is hypermotile relative to the wild type (WT), and treatment with 1 mM cell-permeable 5′-*O*-butyryl 2′,3′-cAMP (Bt-cAMP) (A) or 5′-*O*-butyryl 2′,3′-cUMP (Bt-cUMP) (B) inhibits its hypermotility. (C) Motility of BW25113 WT cells is not impacted by 2′,3′-cNMP hydrolysis through CNPase expression relative to a control strain expressing CNP-inact. (D) FliC expression is unaltered in a Δ*rna* mutant treated with 1 mM Bt-cAMP or Bt-cUMP; the “#” symbol indicates that *fliC* transcript levels were undetectable in the untreated and treated WT cells. (E) Biofilm production is increased in the Δ*rna* mutant relative to that in WT, and treatment with 500 μM Bt-cAMP impairs hyperbiofilm formation. Biofilm was quantified using Congo red staining. (F) Hydrolysis of 2′,3′-cNMPs by CNPase enhances cell survival during acid stress compared to that of a control strain expressing CNP-inact. All data shown are representative of 3 biological replicates; error bars represent ±1 standard deviation. (A and B) Statistical significance was determined by a one-way analysis of variance (ANOVA) with Bonferroni and Holm used as *post hoc* tests to determine significance between samples. A *P* value of <0.01 was considered statistically significant. (C, E, and F) Statistical significance was determined by Student’s *t* test (*, *P* < 0.05; **, *P* < 0.01; ***, *P* < 0.001).

To compare the phenotypic consequences of the altered motility gene expression profiles, we assayed the effect of 2′,3′-cNMPs on flagellum-dependent swimming motility. In agreement with the increased expression of chemotaxis and motility genes in the Δ*rna* mutant relative to that in the WT, previous work had demonstrated that the RNase I-deficient mutant was hypermotile (30) (Fig. 2A and B). However, hydrolysis of 2′,3′-cNMPs in WT cells expressing CNPase does not alter cell motility significantly compared to that of WT cells expressing CNP-inact, indicating that the increased expression of *fliR* and *fliE* is insufficient to confer hypermotility in the presence of RNase I (Fig. 2C). It is of note that the E. coli WT strain BW25113 is known to have poor motility, and quantitative Western blot analysis demonstrated that flagellin (FliC) levels, which reflect late-stage flagellar synthesis, are undetectable in this strain (26); the baseline low level of flagellar biosynthesis may account for the lack of detectable transcriptomic or phenotypic effects by CNPase (31). In contrast, the Δ*rna* strain exhibits enhanced FliC levels compared to those of the WT parent strain (26).

While CNPase is a useful tool for examining phenotypes that arise from depleting 2′,3′-cNMP pools, we also developed methods that allow us to increase 2′,3′-cNMP levels and probe the effects in the E. coli Δ*rna* strain, which produces flagella and is motile. Cells were treated with 5′-*O*-butyryl 2′,3′-cAMP (Bt-cAMP), 5′-*O*-butyryl 2′,3′-cUMP (Bt-cUMP), or 5′-*O*-butyryl 2′,3′-cGMP (Bt-cGMP), which were synthesized based on existing lipophilic 3′,5′-cAMP and -cGMP ester analogs used in eukaryotic systems to facilitate access of the cyclic nucleotides into the cytoplasm (described in the supplemental material) (32). The addition of the cell-permeable cyclic nucleotide derivative Bt-cGMP to the growth medium resulted in observable increased 2′,3′-cGMP levels (Fig. S1), which are comparable to the endogenous level in WT E. coli at mid-exponential growth (24); Bt-2′,3′-cGMP was chosen to quantify permeability and hydrolysis of the butyryl group due to the low limits of quantification and detection. Addition of Bt-2′,3′-cAMP resulted in attenuated motility of the E. coli Δ*rna* strain in a dose-dependent manner without affecting the motility of the WT control strain (Fig. 2A and B), demonstrating that 2′,3′-cNMPs modulate motility in a hypermotile E. coli strain deficient for endogenous 2′,3′-cNMPs. Importantly, neither Bt-cAMP nor Bt-cUMP inhibited cell growth (Fig. S2A and B), and sodium butyrate (NaBt), the product of 5′-*O*-ester hydrolysis, did not alter swimming motility (Fig. S3). Motility was additionally assayed in the presence of 5′-*O*-benzoyl 2′,3′-cUMP (Bz-cUMP) to further probe the utility of cell-permeable 2′,3′-cNMP derivatives with diverse 5′-*O*-ester moieties (see the supplemental material). This compound equally impaired the motility of Δ*rna* (Fig. S4), further validating the role of 2′,3′-cNMPs in this process and ruling out possible confounding effects from the 5′-*O*-ester substituent.

To further probe the molecular basis for how increased 2′,3′-cNMP levels alter the motility of the Δ*rna* strain, the abundance of FliC was quantified by Western blot analysis in WT and Δ*rna* strains grown in the presence and absence of Bt-cAMP. The Δ*rna* strain exhibited a hyperflagellated phenotype, as previously described (26), but treatment with Bt-cAMP and Bt-cUMP did not result in decreased FliC levels (Fig. 2D), despite their inhibitory effects on motility (Fig. 2A and B). These findings demonstrate that exogenously increasing 2′,3′-cNMP concentrations impacts motility without altering the flagellar biosynthetic pathway that leads to FliC expression, indicating that 2′,3′-cNMPs attenuate motility in the Δ*rna* mutant through alternative regulatory mechanisms such as modulation of chemotaxis signal transduction.

### 2′,3′-cNMPs modulate biofilm formation.

Our previous work demonstrated that the Δ*rna* mutant strain displays a hyperbiofilm phenotype due to increased expression of the Curli fiber genes *csgBAC* (24); Curli is the major proteinaceous component of the extracellular matrix generated by E. coli and other members of the Enterobacteriaceae family (33). Biofilm production was likewise increased in WT cells expressing CNPase (24). The RNA-seq results for transcriptomic changes in cells expressing CNPase indicates that depletion of 2′,3′-cNMPs causes upregulation of Curli assembly gene *csgC* (3.74-fold) and type IV pilin assembly gene *hofC* (2.06-fold), along with several Yad fimbrial adhesion genes (*yadCKLMN*) (Table 1). These data provide insight into the transcriptional mechanisms underlying the increased biofilm production upon CNPase-mediated 2′,3′-cNMP hydrolysis and demonstrate that 2′,3′-cNMP depletion is sufficient to increase Curli gene expression, even in the presence of RNase I.

Intriguingly, CNPase expression induced transcription of the *pgaABCD* operon responsible for poly-*N*-acetyl-β-1,6-d-glucosamine (PNAG) biosynthesis in RNase I^+^ E. coli (Table 1), whereas an Δ*rna* mutation dampened expression of the PNAG biosynthetic cluster (24). Transcription from the *pga* promoter is repressed by OmpR (34), and CNPase expression resulted in a modest decrease in *ompR* transcript abundance (1.8-fold), while *rna* deletion did not affect the *ompR* mRNA level (26). These data suggest that the differential modulation of PNAG biosynthesis in WT cells expressing CNPase versus Δ*rna* cells could be regulated, at least in part, by OmpR.

To further evaluate the functional role of 2′,3′-cNMPs in biofilm formation, cultures of WT and Δ*rna* strains were treated with Bt-cAMP. Relative to untreated control cultures, the Δ*rna* mutant produced ∼30% less biofilm in the presence of Bt-cAMP, while WT biofilm formation was unaffected (Fig. 2E), supporting the conclusion that 2′,3′-cNMPs regulate biofilm formation independently of RNase I. As mentioned above, Bt-cAMP did not affect the growth of either strain (Fig. S1). Moreover, sodium butyrate (NaBt) did not perturb biofilm formation (Fig. S5), demonstrating that the observed biofilm inhibition in Δ*rna* results from exogenously increasing the intracellular 2′,3′-cAMP concentration in E. coli lacking both endogenous 2′,3′-cNMPs and RNase I.

### 2′,3′-Cyclic phosphate-containing molecules regulate acid resistance.

Expression of CNPase upregulates transcription of multiple genes involved in acid resistance systems (35, 36), including the genes for glutamate decarboxylase *gadA* (3.91-fold) and *gadB* (7.44-fold), glutamate/gamma-aminobutyric acid antiporter *gadC* (4.35-fold), arginine/agmatine antiporter *aidC* (2.68-fold), lysine decarboxylase *cadA* (3.34-fold), *cadC* transcriptional activator for *cadBA* (3.28-fold), acid stress chaperones *hdeB* (6.00-fold) and *hdeA* (4.78-fold), and acid resistance membrane protein *hdeD* (4.28-fold) (Table 1). The acid tolerance of WT cells expressing CNPase or CNP-inact was evaluated, and the data revealed a 10-fold increase in the survival rate at pH 2.5 upon CNPase expression compared to that of cells expressing CNP-inact (Fig. 2F), suggesting that E. coli acid tolerance is enhanced by the regulatory impact of reducing the levels of 2′,3′-cNMPs and/or oligoribonucleotides with 3′-terminal 2′,3′-cyclic phosphate (26). Our previous work demonstrated that *rna* gene deletion reduced transcript levels of *gadX* and *gadY*, which encode positive transcription regulators of the acid response genes *gadAB* and *gadC* (37), and concomitantly decreased survival in acidic medium relative to that of the WT strain (26). The differences in the acid resistance phenotype and regulation of genes involved in acid tolerance in the Δ*rna* strain versus the CNPase-expressing WT strain may reflect disparate effects of RNase I depletion versus 2′,3′-cNMP hydrolysis, or it may indicate that the complex regulatory network for acid stress response includes the other physiologically relevant CNPase substrate, oligoribonucleotides with 3′-terminal 2′,3′-cyclic phosphates (38, 39).

### 2′,3′-cNMP generation and physiological impacts in Salmonella Typhimurium.

Like E. coli, *S*. Typhimurium is a member of the Gram-negative *Enterobacteriaceae* family. E. coli and *S.* Typhimurium diverged from a common ancestor approximately 120 million years ago (40); phenotypic differences between these species are not only due to genetic differences, including >800 E. coli genes that are absent in the *S.* Typhimurium genome and >1,100 *S.* Typhimurium genes that are absent in the E. coli genome, but also due to differential regulation of conserved genes (41). Due to these two organisms’ evolutionary closeness yet significant phenotypic differences, *S.* Typhimurium was chosen as the first candidate for comparison in order to determine whether bacteria have universal mechanisms for generating 2′,3′-cNMPs and if there are physiological commonalities when 2′,3′-cNMP pools are altered.

**FIG 3:**
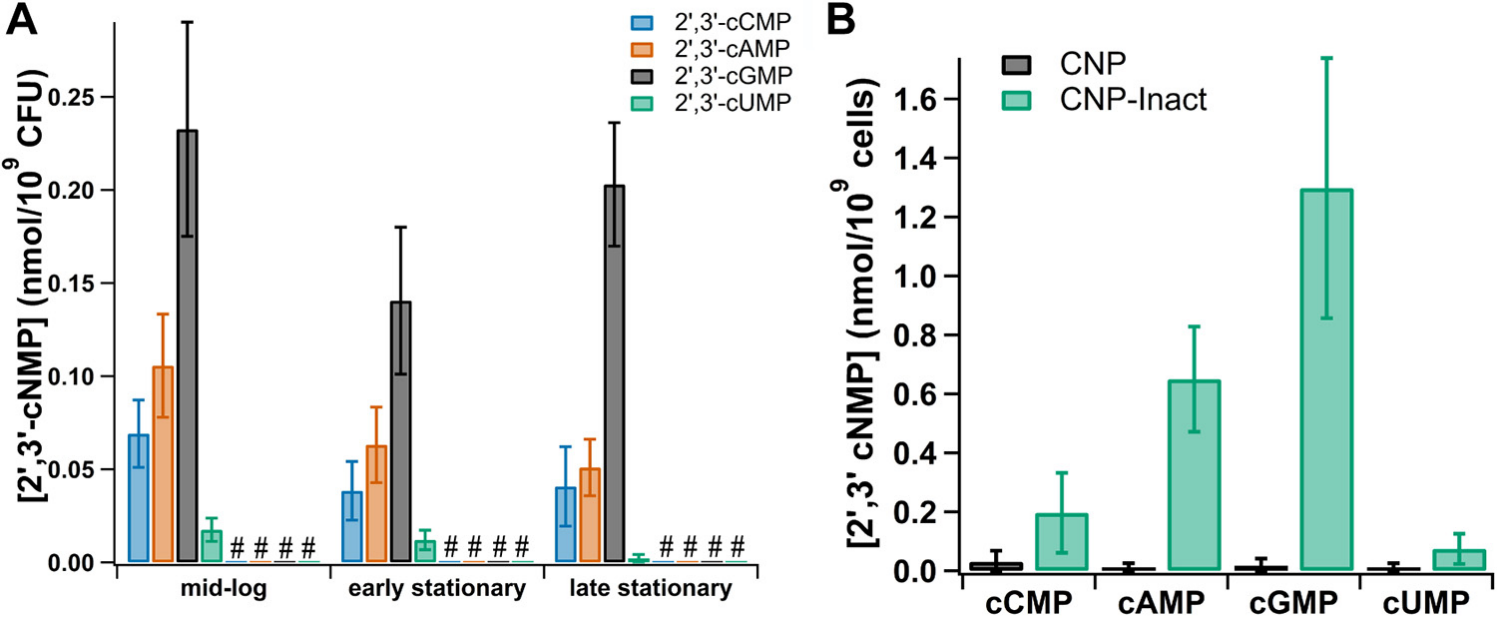
Quantification of 2′,3′-cNMPs in Salmonella Typhimurium. 2′,3′-cAMP, -cGMP, -cCMP, and cUMP levels were quantified by liquid chromatography-tandem mass spectrometry (LC-MS/MS) analysis. (A) WT or Δ*rna* cells were harvested at the mid-log (optical density at 600 nm [OD_600_] = 0.5), early stationary (OD_600_ = 0.9), and late stationary phases (24 h after the onset of growth) to determine growth phase-dependent fluctuations in 2′,3′-cNMP levels. The “#” symbol represents 2′,3′-cNMP levels that were below the level of detection in the Δ*rna* strain. None of the differences in cyclic nucleotide levels between time points were statistically significant. (B) WT cells expressing CNPase or CNP-inact from pKT-CNP or pKT-CNP-inact were harvested at the mid-log phase for transcriptome sequencing (RNA-seq) analysis. Analysis confirmed depletion of all four 2′,3′-cNMPs when CNPase was expressed. Only the levels of 2′,3′-cAMP showed statistical significance when CNPase was expressed, with a *P* value of <0.05. All 2′,3′-cNMP levels are representative of 3 biological replicates; error bars represent ±1 standard deviation. All data shown are representative of 3 biological replicates. Statistical significance was determined by a one-way ANOVA with Bonferroni and Holm used as *post hoc* tests to determine significance between samples.

To determine whether 2′,3′-cNMP levels fluctuate in *S.* Typhimurium in a growth phase-dependent manner, samples were collected at the mid-log, late log/early stationary, and late stationary phases during growth in M9 medium. 2′,3′-cAMP, -cGMP, -cCMP, and -cUMP were most abundant during logarithmic growth, as was seen in E. coli (24); however, levels of all of the nucleotides decreased over the growth curve in *S.* Typhimurium (Fig. 3A). The variation in 2′,3′-cNMP levels at different growth phases was at most ∼5-fold, which is in stark contrast to the approximately 1,000-fold drop in the four 2′,3′-cNMP concentrations in E. coli during early stationary growth. Like E. coli, *S.* Typhimurium expresses an RNase I homolog encoded by the *rna* gene. RNase I is essential for the generation of 2′,3′-cNMPs in E. coli (24); to determine whether the same is true for *S.* Typhimurium, 2′,3′-cNMPs were quantified in an Δ*rna* strain under the same growth conditions as the WT strain. The cyclic nucleotides were below the level of detection at all stages of growth in the *S.* Typhimurium Δ*rna* strain, confirming that 2′,3′-cNMP generation in *S.* Typhimurium is likewise facilitated by RNase I (Fig. 3A).

The effects of 2′,3′-cNMPs on modulation of gene expression in *S.* Typhimurium were probed using RNA-seq analysis with *S.* Typhimurium 14028s WT strains expressing CNPase from pKT-CNP or CNP-inact from pKT-CNP-inact under growth conditions identical to those used for the E. coli RNA-seq analysis. Quantification of 2′,3′-cNMPs by liquid chromatography-tandem mass spectrometry (LC-MS/MS) for the three biological replicates of each strain used in the RNA-seq analysis confirmed that the 2′,3′-cNMP levels were significantly reduced when cells expressed active CNPase compared to those in the inactive variant (Fig. 3B). Comparative analysis of RNA-seq values for the CNPase versus CNP-inact strains indicated that 155 genes were >2-fold downregulated and 14 genes were >2-fold upregulated when active CNPase was expressed (correlating to a decrease in 2′,3′-cNMP levels) (Data Set S2). The overall number of dysregulated genes and the functions of many of those genes differed significantly between *S.* Typhimurium and E. coli expressing CNPase (Fig. 1 and Table 1). Dysregulated genes in *S.* Typhimurium included multiple genes involved in extracellular polysaccharide production, flagellar assembly, heat shock response, peroxide stress response/SOS response, and RNA repair (Table 1); the physiological impacts of the dysregulation of these gene are addressed below. Approximately half of the 155 downregulated genes in *S.* Typhimurium expressing CNPase (*n *= 82) were genes carried by the prophages Gifsy-1, Gifsy-2, Gifsy-3, and ST64B, suggesting a potential role for 2′,3′-cNMPs in regulation of prophage gene expression that is activated by nucleic acid damage (42–44). This is consistent with the CNPase-dependent downregulation of genes involved in responses to nucleic acid damage, i.e., SOS response and RNA repair genes listed in Table 1 (45, 46).

**FIG 4:**
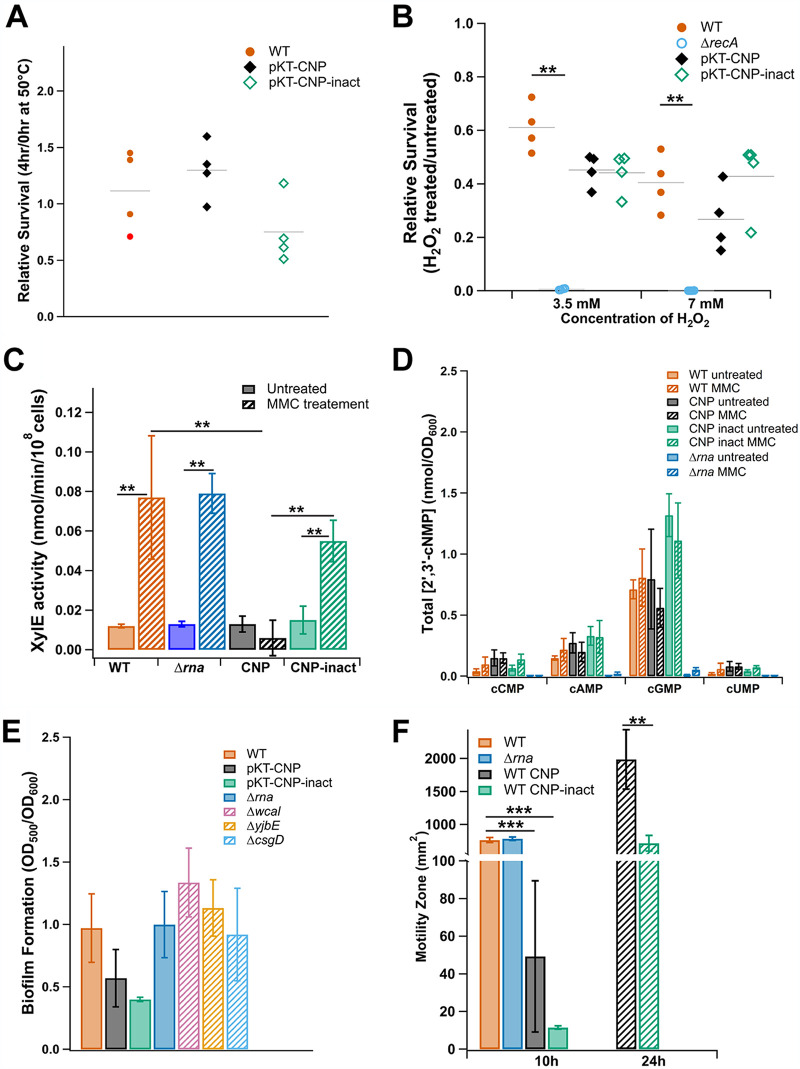
Physiological impacts of depletion of 2′,3′-cNMPs in *S.* Typhimurium. Heat shock survival (A) and peroxide stress survival (B) assays do not reveal significant differences between strains expressing CNPase versus CNP-inact. For heat shock survival, cells were transitioned from 30°C to 50°C and assessed after 4 h. Survival is denoted as CFU per milliliter upon completion of the high temperature treatment divided by CFU per milliliter of the culture at the time of the transition. For peroxide stress, survival is denoted as CFU per milliliter after 2 h of treatment with 3.5 mM or 7 mM H_2_O_2_ relative to that of an untreated control. An Δ*recA* strain was used as a control for H_2_O_2_ sensitivity. Each dot denotes one biological replicate with 3 technical replicates; gray bars denote the mean. (C) Activation of the *rsr-yrlBA-rtcBA* RNA repair operon is impaired during expression of CNPase from pKT-CNP but not in an Δ*rna* mutant. Reporter strains were generated such that XylE activity is directly proportional to the level of activation of the RNA repair operon promoter. Cells were treated with mitomycin C (MMC) as an activating condition or left untreated as a control. Data are representative of 5 biological replicates with 2 technical replicates. (D) 2′,3′-cNMP levels were not significantly altered by MMC treatment. The designated strains, 14028s WT with or without pBAD33-CNP/pBAD33-CNP-inact or Δ*rna*, were treated with MMC or were left untreated for comparison prior to 2′,3′-cNMP extraction and quantification by LC-MS/MS. (E) There was no significant difference in exopolysaccharide (EPS) production/biofilm formation when comparing strains expressing CNPase or CNP-inact, as quantified by Congo red staining. (F) Expression of either CNPase or CNP-inact from the pKT vectors significantly reduced WT motility relative to that of the WT without the plasmid; the Δ*rna* strain did not exhibit significantly altered motility compared to that of the WT. Except where otherwise stated, all data are representative of at least 3 biological replicates; error bars represent ±1 standard deviation. Statistical significance was determined by a one-way ANOVA with Bonferroni and Holm used as *post hoc* tests to determine significance between samples. A *P* value of <0.001 (***) or <0.01 (**) was considered statistically significant.

Only two specific genes showed consistent CNPase-dependent dysregulation in E. coli and *S.* Typhimurium, and both were related to the heat shock response. These were *dnaJ* (downregulated 2.15-fold in E. coli and 3.10-fold in *S.* Typhimurium), which encodes a member of the Hsp40 family of cochaperones that, with the chaperone DnaK, is essential for recovery from heat shock (47, 48), and *ibpB* (downregulated 2.60-fold in E. coli and 7.95-fold in *S.* Typhimurium), which encodes a subunit of the IbpAB small heat shock protein complex (49) (Table 1). The following additional genes related to the heat shock response were downregulated in each species: *dnaK* (2.53-fold) and *ybbN* (2.05-fold) in E. coli and *ibpA* (7.89-fold) in *S.* Typhimurium (Table 1). Together, these data may suggest a role for 2′,3′-cNMPs in regulating the production of certain heat shock chaperones. To determine whether reduction of 2′,3′-cNMP levels influences the heat shock response in *S.* Typhimurium, recovery from heat shock stress was assayed for 14028s WT, CNPase, and CNP-inact expression strains. In addition, survival of peroxide-induced oxidative stress was assayed, as it was recently shown that DnaJ interacts with the RNA polymerase binding partner DksA in a redox-dependent manner during oxidative stress, and deletion of *dnaJ* increases susceptibility of *S.* Typhimurium to hydrogen peroxide-mediated killing (50). The results of the heat shock and oxidative stress survival assays indicate that the level of decreased expression of *dnaJ* and *ibpAB* in CNPase-expressing *S.* Typhimurium is not sufficient to significantly impact survival of heat shock or peroxide-induced oxidative stress (Fig. 4A and B).

The genes most affected by CNPase activity in *S.* Typhimurium were genes of the RNA repair operon (*rsr-rtcB-rtcA*), which were significantly downregulated, as follows: *rsr* (12.75-fold), *rtcB* (13.94-fold), and *rtcA* (14.51-fold) (Table 1). This operon is transcriptionally controlled by RtcR, a member of the bacterial enhancer-binding protein family of regulators that becomes activated in the presence of nucleic acid-damaging conditions, e.g., treatment with mitomycin C (MMC) or hydrogen peroxide (45). Expression of the RNA repair operon of E. coli (*rtcB-rtcA*) was not significantly altered by CNPase activity; this result is consistent with previous work demonstrating that the regulation of the RNA repair operons in E. coli and *S.* Typhimurium responds to different stress conditions (45). To further assess expression of the *S*. Typhimurium RNA repair operon when intracellular levels of 2′,3′-cNMPs are reduced by CNPase, promoter activity was measured using a previously developed reporter assay that utilizes a transcriptional fusion of the RNA repair operon promoter with *xylE* (14028s Δ*rsr*::*xylE*) (45). RtcR-dependent expression of the RNA repair operon was induced by treatment with MMC. The WT reporter strain (14028s Δ*rsrI::xylE*) showed a 6-fold increase in RNA repair operon expression compared to that in the untreated strain, consistent with previously published results (Fig. 4C) (45). Results were similar when strains expressed the CNP-inact variant; conversely, when active CNPase was expressed, XylE activity was reduced to nearly the same level as that in untreated cells (Fig. 4C), confirming that the catalytic activity of CNPase significantly impacts expression of the RNA repair operon in *S.* Typhimurium. This was additionally confirmed for WT 14028s using reverse transcription-quantitative PCR (qRT-PCR) to quantify the levels of *rtcB* transcript; the same trends emerged, showing 12-fold and 10.5-fold increases in *rtcB* transcript levels in the MMC-treated WT and CNP-inact expression strains, respectively, that was abolished in the CNPase expression strain (Fig. S6). These results indicate that CNPase degrades a signal for activation of RtcR, thereby inhibiting initiation of transcription of the RNA repair operon; however, surprisingly, XylE expression in a Δ*rna* reporter strain (14028s Δ*rna* Δ*rsr*::*xylE*) that eliminates production of 2′,3′-cNMPs (Fig. 3A) was similar to WT levels (Fig. 4C).

To determine whether there is potentially another source of 2′,3′-cNMPs during this treatment in *S.* Typhimurium, 2′,3′-cNMP levels were quantified in MMC-treated and untreated cells. The concentration of each of the 2′,3′-cNMPs in the Δ*rna* strain remained near the limit of detection for both treated and untreated cultures (Fig. 4D). Additionally, there was no significant difference in 2′,3′-cNMP levels when comparing MMC-treated versus untreated cultures in the WT, WT pBAD33-CNP, or pBAD33-CNP-inact strains (Fig. 4D). Unlike the dramatic reduction in 2′,3′-cNMP levels seen with expression of CNPase from pKT-CNP (Fig. 3B), expression of CNPase from the pBAD33-CNP plasmid (induced by 0.02% arabinose) did not significantly decrease 2′,3′-cNMP levels (Fig. 4D); however, this same level of CNPase expression from pBAD33-CNP significantly reduced RtcR-dependent expression of the RNA repair operon (Fig. S6). These results indicate that activation of the RNA repair operon in *S.* Typhimurium cannot be clearly linked to fluctuating 2′,3′-cNMP levels. We attempted to further explore whether increasing the 2′,3′-cNMP levels would activate expression of the RNA repair operon in the absence of nucleic acid damage; to this end, the reporter strain (14028s Δ*rsr*::*xylE-kan*) was treated with cell-permeable Bt-cAMP, and expression of the operon was measured at multiple time points over a 25-h period to allow time for intracellular hydrolysis of the butyryl group from Bt-cAMP to release 2′,3′-cAMP (Fig. S7). At the 1.5-, 3-, 6-, and 10-h time points, expression of the operon was unchanged for cultures treated with Bt-cAMP, 2′,3′-cAMP, sodium butyrate, or water; at the 15- and 25-h time points, expression increased nearly 10-fold, but this increase was independent of the treatment and is likely due to cellular stress in the late stationary phase (>12 h after reaching the stationary phase) (Fig. S7). Since Bt-cAMP uptake and hydrolysis to 2′,3′-cAMP in *S.* Typhimurium is likely to be similar to that shown for Bt-cGMP in E. coli (Fig. S1), these results support the conclusions that altered 2′,3′-cNMP levels are not the primary signal for RtcR activation in *S.* Typhimurium and the CNPase substrate that effects RtcR activation and expression of the RNA repair operon is an oligoribonucleotide with a 3′-terminal 2′,3′-cyclic phosphate, which is further addressed below.

Eight genes of the *wca* colanic acid biosynthesis operon were downregulated in *S.* Typhimurium expressing active CNPase (ranging from 2.77-fold to 4.39-fold) (Table 1). Colanic acid is an extracellular polysaccharide (EPS) whose function is not well understood, though research has suggested a role in biofilm or capsule formation and maintenance of the transmembrane electrical potential in the environment (51). Two additional genes, *yjbE* and *yjbF*, which are involved in the synthesis of noncolanic acid EPS, were also downregulated (2.98-fold and 2.40-fold, respectively) (Table 1). Both of these EPS pathways were recognized as part of the Rcs regulon (51), suggesting that reduction of 2′,3′-cNMPs may impact Rcs-mediated EPS biosynthesis. A Congo red assay was performed to determine whether EPS levels are impacted during expression of CNPase in S. Typhimurium. The strains expressing CNPase or CNP-inact showed reduced levels of EPS compared to those in the WT strain, with the inactive variant exhibiting a significant decrease of 2.4-fold; however, the EPS levels were not significantly different between the strains expressing the active and inactive CNPase variants, which suggests that the 2′,3′-cNMP pools did not substantially alter EPS biosynthesis (Fig. 4E). A variety of single-gene deletion mutant controls (Δ*rna*, Δ*wcaI*, Δ*yjbE*, and Δ*csgD*, a regulator of biofilm formation) additionally showed no significant change in EPS levels compared to those of the WT (Fig. 4E); this indicates that, under the growth conditions used, the quantifiable levels of EPS do not significantly change when expression of a single biosynthetic pathway is altered, although the composition of the EPS could potentially be affected.

Of the 14 genes that were upregulated by CNPase, the following four are related to flagellar biosynthesis: *flgBCD*, which are components of the flagellar basal body (5.09-, 5.04-, and 4.40-fold, respectively), and *flgJ*, which encodes a flagellum-specific peptidoglycan hydrolase (3.04-fold) (Table 1). As noted above, CNPase likewise affected the expression of genes related to the flagellar basal body in E. coli. To determine if a motility phenotype could be observed in *S.* Typhimurium, the same swimming motility assay was performed, although, unlike the E. coli parent strain, 14028s is highly motile. The Δ*rna* strain was included to determine if complete abolition of 2′,3′-cNMPs results in a hypermotile phenotype, as observed in E. coli. In these assays, at the 10 h time point, the WT and Δ*rna* strains exhibited equivalent motility, but the WT strains expressing either CNPase or CNP-inact showed significantly reduced motilities relative to that of the WT strain without the plasmids (*P* < 0.001) (Fig. 4F). At the 24-h time point, motility halos could not be measured for the WT and Δ*rna* strains that did not contain the CNPase/CNP-inact plasmids because the halos had already reached the edges of the motility plate; the WT strain expressing CNP-inact exhibited reduced motility relative to that of the strain expressing the active CNPase (*P* < 0.01) (Fig. 4F). Overall, these results indicate that reduction in 2′,3′-cNMP levels by deletion of RNase I does not impact flagellar motility in *S*. Typhimurium and that CNPase hydrolysis of its cellular substrates also does not directly affect motility, since expression of either active CNPase or CNP-inact reduced motility. The effects of the pKT-CNP and pKT-CNP-inact expression plasmids on cell growth are addressed below. Based on RNA-seq data, no *S.* Typhimurium genes related specifically to chemotaxis, rather than flagellar assembly, were altered in their expression by CNPase activity (Data Set S2), which could explain why motility is affected differently in E. coli versus *S.* Typhimurium.

Taken together, these data demonstrate that 2′,3′-cNMPs and other RNA oligomers with terminal 2′,3′-cyclic phosphates differentially affect both transcriptional regulation and physiology of even closely related species. This suggests that different organisms may monitor their intracellular levels of 2′,3′-cNMPs and other 2′,3′-cyclic phosphate-containing molecules and modulate different behaviors in response to this input.

### Effects of hydrolysis of CNPase substrates other than 2′,3′-cNMPs.

It has previously been demonstrated that CNPase cleaves the terminal cyclic phosphate on oligoribonucleotides in addition to targeting cyclic nucleotide monomers (38, 52); both of these CNPase substrates are physiologically relevant in bacteria (39). To determine whether the effects of CNPase on the E. coli and Salmonella transcriptomes are due to 2′,3′-cNMPs vrsus 2′,3′-cyclic phosphates on oligoribonucleotides, we performed RNA-seq of E. coli Δ*rna* and *S.* Typhimurium Δ*rna* strains expressing either CNPase or CNP-inact and compared the results to those from WT-CNPase versus WT-CNP-inact.

Analysis of the transcriptomes for E. coli Δ*rna* expressing CNPase and CNP-inact revealed 178 differentially expressed genes, of which 38 are also dysregulated by CNPase expression in the WT strain (Data Set S3), with 33 genes exhibiting similar dysregulation; 13 of these 33 genes are involved in multiple acid stress response systems (Table 1). This result suggests that CNPase-mediated hydrolysis of RNA oligomers with 2′,3′-cyclic phosphate termini rather than 2′,3′-cNMPs is responsible for increased expression of acid stress response genes (Table 1) and a higher level of acid resistance (Fig. 2F) in the WT strain expressing CNPase. It should be noted that this result is consistent with our previously reported analysis of the E. coli Δ*rna* strain, which showed decreased expression of *gadX* and *gadY* of the glutamate-dependent acid resistance system and decreased acid resistance (26); there are a number of regulatory circuits for the various acid tolerance systems (53) that could be differentially responsive to 2′,3′-cyclic phosphate-containing molecules. Overall, this analysis of the transcriptomes for E. coli Δ*rna* strains expressing CNPase and CNP-inact demonstrates that only 38 of the 519 genes identified as dysregulated by CNPase expression in the WT strain are dysregulated by CNPase in the absence of 2′,3′-cNMPs (Data Set S3).

In the *S.* Typhimurium Δ*rna* strain, 669 genes exhibit CNPase-dependent differential gene expression, but only 27 of these genes are similarly dysregulated by CNPase expression in the WT strain, which include the flagellar assembly genes *flgB*, *flgC*, *flgD*; RNA repair genes STM14_4239, *rtcB*, and *rtcA*; heat shock response genes *dnaJ*, *ibpB* and *ibpA*; and SOS response genes *recN and yebG* (Table 1; see also Data Set S4 in the supplemental material). The CNPase-dependent differential expression of these 27 genes in both the presence (WT strains) and absence (Δ*rna* strains) of 2′,3′-cNMPs suggests that oligoribonucleotides with 3′-terminal 2′,3′-cyclic phosphates are involved in their regulation. Recent work by Hughes et al. (54) shows that the regulatory ligand that activates RtcR, thereby stimulating transcription of STM14_4239, *rtcB*, and *rtcA* from the RNA repair operon promoter, is a tRNA fragment ending with a 2′,3′-cyclic phosphate. The CNPase-dependent dysregulation of the RNA repair operon in both the WT and Δ*rna* strains, as well as the results of our RtcR activation assays (Fig. 4C and Fig. S6 and S7), are consistent with CNPase-mediated cleavage of the 2′,3′-cyclic phosphate moiety of these cleaved tRNAs preventing activation of RtcR. An additional 13 genes exhibited dysregulation in both Δ*rna*-CNP/inact and WT-CNP/inact strains, but with opposite regulatory effects; these included mostly prophage genes (Data Set S4). RNA-seq analysis of *S.* Typhimurium WT and Δ*rna* strains without the CNPase/CNP-inact expression plasmids revealed 149 genes that are differentially expressed in the absence of RNase I (Data Set S5); none of these 149 genes overlap the gene set that is dysregulated by CNPase expression in both the WT and Δ*rna* strains. This result supports that the CNPase substrate responsible for the altered expression of the RNA repair genes, as well as the other similarly dysregulated genes between the WT and Δ*rna* CNPase/CNP-inact strains (Table 1 and Data Set S4), is an oligoribonucleotide with a 3′-terminal 2′,3′-cyclic phosphate.

To further assess physiological effects of CNPase or CNP-inact expression from the pKT vectors in E. coli and *S.* Typhimurium, growth curves were performed with both WT and Δ*rna* strains (Fig. S8); the Fig. S8 legend includes the calculated doubling times and statistical analyses. For E. coli and *S.* Typhimurium WT and Δ*rna* strains, there was a small, but significant, increase in the doubling times for all but one of the strains expressing CNPase or CNP-inact relative to those for the strains without the pKT vectors. However, there was no significant difference between the doubling times for strains expressing CNPase and CNP-inact. These results indicate that the effects of the pKT-CNP or pKT-CNP-inact plasmids on E. coli and *S*. Typhimurium growth rates are independent of the catalytic activity of CNPase. In addition, the approximately 2-fold reduced growth rate for WT *S.* Typhimurium expressing CNPase or CNP-inact relative to that of the WT strain is consistent with the reduced flagellar motility for the WT strain expressing either CNPase or CNP-inact (Fig. 4F); since both the active and inactive CNPase affect *S.* Typhimurium growth and motility, hydrolysis of 2′,3′-cyclic phosphates does not cause the phenotype. The effect of CNPase/CNP-inact on E. coli and *S*. Typhimurium growth may be due to binding of substrate molecules, thereby preventing their regulatory activities or turnover. Consistent with this hypothesis, CNPase and CNP-inact exhibit the same *K_m_* values but very different *k*_cat_ values (27); in addition, CNPase has been shown to bind eukaryotic poly(A)^+^ mRNA independent of its catalytic activity (29).

### 2′,3′-cNMPs in other organisms.

Using literature review along with metabolomics data within a number of publicly available databases, 2′,3′-cNMPs were identified in data sets from Staphylococcus aureus, Pseudomonas fluorescens, Mus musculus, and Arabidopsis thaliana, as well as within human tissues (Table 2). All four of the 2′,3′-cNMPs were not reported in each data set; however, the data were often from untargeted metabolomics runs where 2′,3′-cNMPs were not specifically under investigation (60, 66, 87). In other cases, only certain 2′,3′-cNMPs were targeted for investigation (17, 21, 64, 65, 85, 88). All of the data sets utilized either tandem mass spectrometry (MS/MS), in which the diagnostic fragments allow unambiguous identification of 2′,3′-cNMPs, or LC-MS (without additional fragmentation), which allows for the identification of 2′,3′-cNMPs due to retention time.

**TABLE 2:** Organisms known to contain 2′,3′-cyclic nucleotide monophosphates

Organism	2′,3′-cNMP(s) detected	Database	Source and/or reference(s)
Bacteria			
Escherichia coli	cAMP, cGMP, cCMP, cUMP		23, 24; this study
Pseudomonas fluorescens	cCMP, cUMP		21
Salmonella Typhimurium	cAMP, cGMP, cCMP, cUMP		This study
*Staphylococcus aureus*	cAMP		85
Animals			
Homo sapiens	cAMP, cGMP, cCMP, cUMP	MW	60, 65, 86
Mus musculus	cAMP, cGMP, cCMP, cUMP	MW	63, 87
Rattus norvegicus	cAMP, cGMP, cCMP, cUMP		20
Rabbit	cAMP, cGMP		65
Plant			
Arabidopsis thaliana	cAMP, cGMP, cCMP		17, 64, 66
*Nicotiana tabacum*	cAMP, cGMP, cCMP		88

## DISCUSSION

Nucleotide signaling mediates responses to various environmental stimuli in diverse bacterial taxa, orchestrating complex processes such as the motility-sessility transition, sporulation, and virulence factor production (7). The present work utilizes chemical and enzymatic perturbation of *in vivo* 2′,3′-cNMP levels in coordination with global transcriptome profiling and phenotypic analyses to characterize the function of 2′,3′-cNMPs in bacterial nucleotide signaling. These studies have identified multiple roles for 2′,3′-cNMPs, as well as for oligoribonucleotides with 2′,3′-cyclic phosphate termini, in bacterial physiology within distinct cellular processes.

While the mechanism through which 2′,3′-cNMPs govern specific cell processes is a current subject of investigation, it is possible that regulation occurs in part through direct interactions of the nucleotides with nucleotide-binding effectors. There is precedence for this hypothesis, as recent work identified the polyadenylate-binding protein Rbp47b in A. thaliana as the first known 2′,3′-cNMP-binding effector in any organism (17). Rbp47b serves as a scaffold for assembly of the multiprotein stress granule complex, and this assembly is facilitated by 2′,3′-cAMP binding to Rbp47b (17). Intriguingly, Rbp47b also binds mRNA (67), suggesting that stress granule formation is modulated by the extent of mRNA decay in the cell, as degradation of poly adenylated mRNA likely increases the 2′,3′-cAMP concentration. These findings allude to the existence of other effectors in diverse organisms that sense RNA-derived 2′,3′-cNMPs as reporters of cellular stress. Another possibility is that 2′,3′-cNMPs play an indirect role in gene regulation by affecting nucleotide metabolism, influencing the concentrations of various nucleotide pools that themselves regulate a subset of genes.

Our previous work demonstrated that, in E. coli, all detectable 2′,3′-cNMPs are generated by the exoribonuclease RNase I (encoded by *rna*). Interestingly, in this work, we found that expressing CNPase to directly target the cyclic nucleotide pools leads to the dysregulation of many transcripts that were not previously found to be perturbed by deletion of *rna* (26), indicating that RNase I elicits cellular effects that are separate from those caused specifically by 2′,3′-cNMP fluctuations. One striking example is the Trp biosynthetic cluster *trpDCBA*, which was ∼5- to 7-fold downregulated in RNase I^+^ E. coli expressing CNPase but was not altered by *rna* gene deletion. A possible explanation for these CNPase-specific effects is altered flux through 2′,3′-cNMP catabolic pathways, as CNPase expression inevitably perturbs other nucleotide/side pools due to 2′,3′-cNMP hydrolysis. In addition, CNPase cleaves terminal phosphates of cleaved regulatory oligonucleotides that affect transcript levels. Conversely, catabolism of 2′,3′-cNMPs does not occur in the Δ*rna* strain, as this strain lacks detectable 2′,3′-cNMP concentrations (24), which likely alters nucleotide salvage pathways.

In addition to serving as key components of cellular metabolism, certain nucleotides are established modulators of bacterial chemotaxis and motility. Here, we identified 2′,3′-cNMPs as a novel component of nucleotide signaling in motility regulation. RNA-seq data link 2′,3′-cNMPs to transcriptional regulation of intracellular components of chemotaxis and flagellar assembly via a failure to generate 2′,3′-cNMPs (Δ*rna*) (Fig. 2F) or via depletion of existing 2′,3′-cNMP pools (CNPase). The effects of CNPase expression on motility-related genes were minor and were echoed in results of a soft agar motility assay, with the WT strain expressing CNPase showed no change in motility. However, the hypermotile Δ*rna* strain partially attenuated motility due to the addition of cell-permeable 2′,3′-cNMP analogs to the medium in a manner that does not affect FliC abundance (Fig. 2A, B, and E). We previously determined that 2′,3′-cNMPs cannot traverse the E. coli cell envelope (24) and that 2′,3′-cAMP has no effect on swimming motility (see Fig. S5 in the supplemental material), indicating that 2′,3′-cNMPs do not function as exogenous chemoattractants or -repellents, as these processes require transport across the outer membrane and subsequent binding to periplasmic methyl-accepting chemotaxis proteins (MCPs) (68). Together, these data suggest that 2′,3′-cNMP levels impact motility at an as-yet-unidentified stage of biosynthesis or chemotaxis, which may become clearer upon repeating these experiments in a more motile strain of E. coli.

Nucleotide metabolism and c-di-GMP signaling govern biofilm production, in addition to regulating flagellar motility. Previous phenotypic assays and global gene expression analyses with WT and Δ*rna* E. coli strains demonstrated that 2′,3′-cNMPs also function in biofilm regulation (24), thus identifying a novel nucleotide pool involved in biofilm formation. The present work provides further insight into the role of 2′,3′-cNMPs in biofilm production through the discovery that decreasing 2′,3′-cNMP levels upregulated expression of Curli assembly gene *csgC* (Fig. 3B), and treatment with Bt-cAMP attenuated the increased biofilm phenotype in RNase I-deficient E. coli (Fig. 3A). Perturbation of 2′,3′-cNMP levels or deletion of the RNase I gene additionally affected transcription of genes involved in nucleotide metabolism, establishing a link to prior studies that indicated that perturbation of *de novo* pyrimidine or purine nucleotide biosynthesis alters biofilm morphology in E. coli (11–13). Indirect modulation of c-di-GMP biosynthesis functions as one mechanistic link between primary nucleotide metabolism and biofilm formation, as antimetabolite-mediated disruption of *de novo* purine synthesis decreases the concentration of c-di-GMP and impairs biofilm production in E. coli (13). Therefore, the finding that disrupting 2′,3′-cNMP concentrations dysregulated the expression of purine and pyrimidine nucleotide metabolic genes suggests that 2′,3′-cNMPs modulate nucleotide pools and can perturb c-di-GMP signaling, which likely mediates the biofilm phenotype in WT cells expressing CNPase. The expression of several genes encoding diguanylate cyclases (DGCs) and c-di-GMP phosphodiesterases (PDEs) also was altered by CNPase expression, further suggesting that 2′,3′-cNMPs influence biofilm production through modulation of distinct c-di-GMP metabolic enzymes. Although previous investigations in our group determined that the total concentration of c-di-GMP is not perturbed in E. coli lacking RNase I (24), modulation of individual DGCs or c-di-GMP PDEs can impact E. coli biofilm production without altering the global abundance of c-di-GMP (69). The emerging regulatory functions for 2′,3′-cNMPs in biofilm formation could inform the discovery of novel adjuvants to remedy chronic, biofilm-associated microbial infections.

Characterization of the generation of 2′,3′-cNMPs in *S. Typhimurium* revealed basic similarities with E. coli, including the essential role of RNase I in production of 2′,3′-cNMPs and the overall highest level of 2′,3′-cNMP expression during mid-logarithmic growth. However, there was little overlap in the cellular responses to altered levels of 2′,3′-cNMPs between these two highly related species; therefore, it is likely that investigation of the roles of 2′,3′-cNMPs in other microbes will reveal diverse regulatory proteins and pathways that respond to 2′,3′-cNMPs. In addition, it is now apparent that oligoribonucleotides with terminal cyclic phosphates also have regulatory activities, as demonstrated for RtcR activation and expression of the RNA repair operon (54), thus expanding the repertoire of cyclic nucleotides that control stress responses of bacteria.

In addition to our identification of 2′,3′-cNMPs in E. coli and *S.* Typhimurium, a search of publicly available metabolomics databases yielded hits for 2′,3′-cNMPs in additional bacterial species, as well as in eukaryotes. While some of the species have an RNase I or RNase T2 homologue, such as C. elegans and humans, not all have a known RNase T2 homologue (M. genitalium), suggesting that other RNase families that use a similar two-step RNA hydrolysis mechanism may serve as the source for 2′,3′-cNMPs. Given previous work demonstrating a link between 2′,3′-cAMP and organ damage (18, 70) in mammals and between 2′,3′-cAMP/2´,3′-cGMP levels and wounding in plants (64), it seems likely that 2′,3′-cNMP levels are broadly used by organisms to modulate their environmental responses.

Here, we identify 2′,3′-cNMP pools as novel components of bacterial signal transduction with implications in pathogenesis, expanding the scope of nucleotide signaling beyond the paradigmatic 3′,5′-cNMPs and c-di-NMPs. Transcriptional profiling and phenotypic investigations, in tandem with controlled manipulation of 2′,3′-cNMP levels, have identified bacterial processes regulated by 2′,3′-cNMPs, providing novel links between nucleotide metabolism and virulence-associated phenotypes such as motility and acid tolerance. Additional gene expression analyses and bioanalytical experiments suggest that these processes are mediated in part by dysregulated nucleotide homeostasis upon perturbation of 2′,3′-cNMP levels. Future experiments aim to identify potential 2′,3′-cNMP-binding effectors and provide additional mechanistic insight into the functions of RNase I and 2′,3′-cNMP pools in prokaryotic physiology. In addition, the recombinant CNPase and cell-permeable 2′,3′-cNMP derivatives developed here will enable dissection of processes linked to 2′,3′-cNMPs and T2 family RNases across the kingdoms of life.

## MATERIALS AND METHODS

### Bacterial strains, plasmids, general culture conditions, commercial chemicals, and statistical analyses.

All relevant strains and plasmids used in this study are listed in Table S1 in the supplemental material. The RNase I-deficient E. coli strain (*rna*::*kan* Δ*rna*) in the BW25113 genetic background (*lacI*^q^ *rrnB*_T14_ Δ*lacZ*_WJ16_ *hsdR514* Δa*raBAD*_AH33_ Δ*rhaBAD*_LD78_) was obtained from the Keio collection, along with the BW25113 WT strain (71, 72). Locus-specific PCR amplifications were used to confirm disruption of the *rna* gene with the *kan* cassette, as reported previously (Table S2, oligonucleotides 1 to 4) (24). The WT *S.* Typhimurium strain used in this study is ATCC 14028s. Strains JEK20 (14028s Δ*rna*) and JEK30 (14028s Δ*recA*) were generated by transduction of the WT strain with HT P22 lysate grown on appropriate single-gene deletion mutants from the BEI collection (described in (73). The *cat* or *kan* cassette, respectively, for selection of transductants was subsequently excised by transforming cells with pCP20 (72), and the deletion was confirmed by PCR with gene-flanking primers (Table S2, oligonucleotides 5 and 6 and oligonucleotides 7 and 8, respectively). Construction of the reporter strains JEK12 (14028s Δ*rsr*::*xylE*-*kan*) and JEK17 (14028s Δ*rsr*::*xylE*) was described previously (45). JEK29 (14028s Δ*rna* Δ*rsr*::*xylE*) was constructed similarly to JEK20, except the recipient strain for P22 transduction was JEK17. Strains 14028s Δ*yjbE*::*kan*, 14028s Δ*wcaI*::*kan*, and 14028s Δ*csgD*::*kan* were obtained from the BEI SGD collection, and deletion-insertion mutations were confirmed by PCR with gene-flanking primers (Table S2, oligonucleotides 9 and 10, 11 and 12, and 13 and 14, respectively).

Bacteria were routinely cultured in Luria-Bertani (LB) medium or M9 minimal medium supplemented with 0.4% glucose and 0.2% Casamino Acids at 37°C with orbital shaking at 200 to 225 rpm, unless otherwise noted. Antibiotics were used at the following working concentrations unless otherwise noted: kanamycin (25 μg/ml), carbenicillin (100 μg/ml), and chloramphenicol (30 μg/ml). Plasmids pKT-CNP and pKT-CNP-inact (H73L/H152L), which encode *bla* for β-lactam-mediated selection, were obtained by restriction enzyme-based subcloning as previously described (24). Plasmids pKT-CNP(*cat*) and pKT-CNP-inact(*cat*), encoding *cat* for chloramphenicol resistance, were constructed using polymerase incomplete primer extension (PIPE) cloning, using either pKT-CNP or pKT-CNP-inact as the vector template and pBAD33 as the template for the *cat* insert (Table S2, oligonucleotides 19 to 22) (74). For *S.* Typhimurium, plasmids pKT-CNP and pKT-CNP-inact were freshly transformed by electroporation prior to each experiment, as plasmids exhibited some toxicity. For qRT-PCR experiments, pBAD33-CNP and pBAD33-CNP-inact were utilized as a less toxic alternative; for these, the Rattus norvegicus CNPase (rCNPase) catalytic domain was codon optimized and synthesized by GenScript (Piscataway, NJ) and subcloned into pET-28a within the NdeI and XhoI restriction sites. rCNPase was subcloned into pBAD33 using NdeI and XhoI for expression to make pBAD33-CNP. pBAD33-CNP-inact (H68L/H147L) was generated using the QuikChange site-directed mutagenesis kit (Agilent, Santa Clara, CA) with the provided primer sequences (Table S2, oligonucleotides 15 to 18).

The sodium salts of nucleoside 5′-monophosphates and nucleoside 5′-diphosphates were purchased from Chem-Impex (Wood Dale, IL) for use as analytical standards. Analytical standards of adenosine 2′,3′-cyclic monophosphate and cytidine 2′,3′-cyclic monophosphate (monosodium salts) were obtained from Carbosynth (Berkshire, UK). Standards of guanosine 2′,3′-cyclic monophosphate and uridine 2′,3′-cyclic monophosphate (monosodium salts) were supplied by Biolog (Bremen, Germany). Adenosine 3′-monophosphate (free acid) and 8-bromo adenosine 3′,5′-cyclic monophosphate (sodium salt) were purchased from Sigma-Aldrich. Uridine 3′-monophosphate (disodium salt) was obtained from Chem-Impex.

Experiments utilized at least three biological replicates (*n* ≥ 3), and a two-sample *t* test was employed to assess statistical significance between two data sets, where equal or unequal variance was evaluated using an *F* test. A *P* value of <0.05 was considered statistically significant. For larger data sets, statistical analysis was assessed through analysis of variance (ANOVA) to determine whether there was statistical significance, followed by *post hoc* Bonferroni and Holm tests to determine which groups were statistically significant. A *P* value of <0.01 was considered statistically significant.

### Transcriptome profiling of WT and Δ*rna* strains of E. coli and *S.* Typhimurium containing pKT-CNP or pKT-CNP-inact and of WT/Δ*rna* for *S*. Typhimurium.

Overnight cultures for three biological replicates each of WT or Δ*rna* strains of E. coli BW25113 or *S.* Typhimurium 14028s containing pKT-CNP or pKT-CNP-inact, or WT and Δ*rna* strains of *S.* Typhimurium 14028s without plasmids, in M9 medium (0.4% glucose and 0.2% Casitone) with appropriate antibiotics were subcultured 1:100 in the same medium and grown at 37°C with shaking. When cultures reached an optical density at 600 nm (OD_600_) of 0.1, expression of CNPase or CNP-inact was induced with 25 ng/ml anhydrotetracycline. Cultures were grown to an OD_600_ of 0.5 to 0.6, and samples were collected for RNA-seq (and 2′,3′-cNMP quantification for the WT strains). Cells were pelleted by centrifugation, flash frozen, and stored at −80°C.

For the WT strains with the pKT plasmids, the frozen cell pellets were sent to the Emory University Yerkes Nonhuman Primate Genomics Core for RNA isolation, rRNA depletion, library construction and sequencing. Briefly, total RNA (2 μg) was extracted and subjected to rRNA depletion using the Bacterial Ribo-Zero rRNA removal kit (EpiCentre, Madison, WI) following the manufacturer’s instructions. Complementary DNA (cDNA) libraries were prepared with the ScriptSeq v2 RNA-seq library preparation kit (Epicentre, Madison, WI, USA). The rRNA-depleted total RNA sample was fragmented using an RNA fragmentation solution, and the fragmented RNA was reverse transcribed into cDNA using random hexamer primers containing a tagging sequence at the 5′ end; 3′ tagging was accomplished using a terminal-tagging oligonucleotide (TTO) consisting of a random hexamer flanked by a 5′ tag sequence and a blocked 3′ terminus. The di-tagged cDNA was purified using AMPure XP beads (Agencourt, Beckmann-Coulter, USA), and PCR amplified to add index and sequencing adapters. After amplification, the final library was purified using AMPure XP beads, and the final pooled libraries were sequenced on the Illumina HiSeq 3000 system in a single-end (SE) 150-cycle format. Each sample was sequenced to approximate depth of 8 to 12 million reads. For data processing and statistical analysis, RNA-seq reads were aligned to the GenBank E. coli BW25113 genomic reference sequence (accession number CP009273.1) or GenBank Salmonella enterica subsp. *enterica* serovar Typhimurium 14028s sequence (CP001363.1) using the STAR aligner v2.5.2b (75), and transcript abundance was estimated using htseq-count v0.6.1p1 (76). Differential expression analysis was performed with DESeq2 (77). DESeq2 uses the Benjamini-Hochberg adjustment of *P* values for false-discovery rate (*P*_adj_); log_2_ fold change values for differential gene expression with *P*_adj_ values of <0.05 were considered significant. The NCBI index (ftp://ftp.ncbi.nlm.nih.gov/genomes/all/GCA/000/750/555/GCA_000750555.1_ASM75055v1) was utilized for gene annotation.

For the Δ*rna* E. coli strains with pKT plasmids, total RNA was isolated from the frozen cell pellets and treated with DNase I using the New England Biolabs (NEB) Monarch total RNA miniprep kit, following the manufacturer’s instructions. For the WT (without pKT plasmids) and Δ*rna* (with and without pKT plasmids) strain *S.* Typhimurium frozen cell pellets, total RNA was isolated and treated with DNase I as previously described (45). Total RNA was sent to Microbial Genome Sequencing Center (MiGS); 300 ng of RNA was used for library preparation with Illumina Stranded Total RNA Prep with Ribo-Zero Plus, following the manufacturer’s protocol. The libraries were run on an Illumina NextSeq 2000 instrument, providing >12 million paired-end reads (2 × 50 bp). MiGS performed analysis as follows. Sequencing data were processed with Illumina bcl2fastq2 conversion software; read mapping and quantification were performed with HISAT2 (78) and the feature “Counts” in the Subread package (79), respectively, using the same reference genomes as described above for the WT strain RNA sequencing. Read counts were normalized using the edgeR (80) trimmed mean of M values (TMM) algorithm. Differential expression analysis was performed using the edgeR quasilinear *F* test (glfTest) functionality against treatment groups; genes with an absolute log_2_ fold change (log_2_FC) of >1 and a *P*_adj_ value (FDR) of <0.05 were considered differentially expressed. Principal-component analysis (PCA) and differentially expressed gene heat maps indicated that one of the three biological replicates for Δ*rna*-CNPase (deltaCNP1) was an outlier in the RNA-seq analysis of E. coli Δ*rna* strains containing pKT-CNP or pKT-CNP-inact. The data for deltaCNP1 were removed from the analysis, and differential gene expression analysis was performed with the data sets for the other two biological replicates of Δ*rna*-CNPase and the three biological replicates for Δ*rna*-CNP-inact (deltai1 to deltai3).

### Growth curves.

Growth curves were performed for E. coli and *S*. Typhimurium WT and Δ*rna* strains with and without the pKT-CNP and pKT-CNP-inact plasmids. For E. coli and *S.* Typhimurium strains, single colonies were inoculated into 3 ml M9 medium (0.4% glucose and 0.2% Casamino Acids) with appropriate antibiotics and were grown at 37°C overnight with shaking. E. coli cultures were diluted 1:100 in 2 ml fresh M9 medium in 24-well culture plates for growth at 37°C with automated OD_600_ readings at 15-min intervals in a Biotek Epoch 2 microplate reader. *S.* Typhimurium cultures were diluted in 25 ml fresh M9 medium to an OD_600_ of ∼0.05 in 250-ml flasks and incubated with aeration at 37°C; samples were taken at 30- to 60-min intervals for OD_600_ readings on a spectrophotometer. At an OD_600_ of ∼0.1 to 0.2, cultures were induced with 25 ng/ml anhydrotetracycline. Cultures were then grown to the late stationary phase. At least 3 biological replicates were performed for each strain. Doubling time (*T_d_*) for each strain was calculated in Excel (Microsoft) using the slope (α) from the linear region of the exponential phase in a semilogarithmic plot of OD_600_ versus time, as follows: *T_d_* = ln(2)/α.

### Motility assay.

Bacterial swimming motility was assayed as previously published, with minor modifications (33). Autoclaved motility medium (1% tryptone, 0.5% NaCl, and 0.3% agar) was cooled in a 60°C water bath. The medium was supplemented with appropriate antibiotics, 25 ng/ml anhydrotetracycline to induce pKT-CNP/pKT-CNP-inact, and/or 1 mM cell-permeable 2′,3′-cNMP derivatives as required for each experiment. The medium was aliquoted into 60-mm × 15-mm petri dishes (10 ml of medium per dish) or 100-mm × 15-mm petri dishes (25 ml/dish). After allowing the plates to solidify overnight, 2.5 μl of bacterial culture (diluted 1:100 from an overnight culture in LB) were inoculated into the surface of each plate. For E. coli, the plates were incubated for 24 h at 30°C; *S.* Typhimurium plates were incubated at 30°C and results recorded at 10 h and 24 h. The area of the motility zone was quantified by measuring the zone across three diameters.

### Quantitative Western blot analysis of flagellar protein expression.

Cultures of WT and Δ*rna* strains (1.5 ml) were grown in 15-ml plastic culture tubes in the presence of Bt-cAMP (1 mM), Bt-cUMP (1 mM), or vehicle (water). Upon reaching an OD_600_ of ∼0.6, 1 ml of culture was harvested by centrifugation at 10,000 × *g* for 5 min at room temperature (RT). The pellet was flash frozen in liquid nitrogen and stored at −80°C. For Western blot analysis, the insoluble protein fraction was isolated by lysing cells into 100 μl of BugBuster (EMD-Millipore), according to the manufacturer’s protocol. Following resuspension of the protein pellet in 100 μl of sodium phosphate buffer (50 mM [pH 7.4]), the protein concentration was normalized across all samples using a Bradford assay (protein assay dye; Bio-Rad). Protein samples were diluted 1:1 with 2× Laemmli buffer, denatured by heating at 95°C for 10 min, and separated by SDS-PAGE on Criterion TGX precast midi protein gels (4 to 20% acrylamide; Bio-Rad) at 4°C. The Trans-Blot Turbo transfer system (Bio-Rad) was used to transfer separated proteins to nitrocellulose membranes (0.2 μm; Bio-Rad) using the “mixed MW” mode, according to the manufacturer’s instructions. The blots were processed essentially as described in the Opti-4CN substrate kit (Bio-Rad). Briefly, blots were blocked by incubation in 3% Blocker solution (Bio-Rad) for 2 h at room temperature, followed by 12 h of incubation at 4°C with rabbit polyclonal anti-flagellin primary antibody (15,000-fold dilution, catalog no. 93713; abcam). The blots then were incubated for 1 h at room temperature with goat anti-rabbit secondary antibody conjugated to horseradish peroxidase (12,000-fold dilution, catalog no. 205718; abcam), and bands were detected by incubation with the Opti-4CN substrate for 15 min at room temperature. Blots were imaged using an Epson Perfection V600 photo scanner operating with the “professional” setting to obtain 16-bit gray-scale TIF image files, and band densitometry was quantified using ImageJ (National Institutes of Health). The identity of the FliC band was confirmed by Western blot analyses using strain BW25113 *fliC*::kan^r^ (Δ*fliC*) (obtained from the Keio collection) (71) as a negative control and strain K-12 W3110 overexpressing FliC from plasmid pCA24N-*fliC* (obtained from the ASKA collection) (81) as a positive control (26).

### Congo red biofilm assay.

The Congo red assay utilized for EPS/biofilm quantification is based on a published protocol (35).

### (i) E. coli.

LB (3 ml) was inoculated with a single colony of BW25113 (WT) or Δ*rna* cells from LB-agar plates and cultured overnight in 15-ml plastic culture tubes. Each overnight culture was used to inoculate (1:50) 10 ml YESCA (1% Casamino Acids and 0.12% yeast extract) containing 0.0025% Congo red in 50 ml Celltreat conical tubes (sterile polypropylene) with the lids left loose for gas exchange. After reaching an OD_600_ of ∼0.3 to 0.4, 1 ml of each culture was transferred to a 1.6-ml Eppendorf tube and either treated with vehicle (YESCA medium) or with 500 μM Bt-cAMP. The cultures were incubated for 24 h at room temperature without shaking (lids were left open and the tubes were loosely covered in plastic wrap and foil). For each culture, 200 μl of supernatant was transferred to a 96-well microplate (Corning Costar) following centrifugation at 12,000 × *g* for 15 min. Biofilm formation was quantified by recording the absorbance at 500 nm (*A*_500_). To normalize for cell density, each culture was disturbed by pipetting, and 200 μl was transferred to a 96-well microplate prior to recording the OD_600_ using a microplate reader. The *A*_500_ for the supernatant was subtracted from the *A*_500_ of the Congo red medium that was used to grow the cultures and then normalized to OD_600_ for the resuspended pellets.

### (ii) *S.* Typhimurium.

The Congo red assay was slightly modified based on optimized conditions for biofilm formation in *S.* Typhimurium (59). A 5-ml aliquot of LB was inoculated with single colonies of 14028s WT, WT (pKT-CNP), WT (pKT-CNP-inact), Δ*rna*, Δ*yjbE*::*kan*, Δ*wcaI*::*kan*, or Δ*csgD*::*kan* strains in 15-ml polypropylene conical tubes and grown overnight at 30°C with shaking. Each overnight culture was normalized to an OD_600_ of 0.8 and subcultured (1:100) into 5 ml dilute tryptic soy broth (TSB) medium (1:20 TSB in deionized water [diH_2_O]) containing 0.0025% Congo red and 25 ng/ml anhydrotetracycline. From each of the inoculated cultures, 1-ml aliquots were transferred into three 1.6-ml microcentrifuge tubes for technical replicates. Tubes were incubated with lids left open (lightly covered in plastic wrap) for 24 h at 30°C with slow shaking (24 rpm). The amount of Congo red that was incorporated into EPS/biofilm was calculated as described above.

### Acid sensitivity assay.

Acid resistance was assayed according to published methodology (36). Upon reaching an OD_600_ of 0.2, cultures of WT, WT pKT-CNP, and WT pKT-CNP-inact (3 ml) were treated with anhydrotetracycline (25 ng/ml) to induce protein expression. Incubation was continued to an OD_600_ of ∼0.6, and the cultures then were inoculated 1:20 into 2 ml of fresh M9 medium (0.4% glucose and 0.2% Casamino Acids) at either pH 2.5 (pH adjusted using HCl) or pH 7 (control) in a 24-well microtiter plate (sterile untreated polystyrene; VWR International). The cultures were incubated for 2 h, and volume-normalized CFU (CFU per milliliter) were quantified by 6 by 6 drop plating (82) to determine the survival rate of each strain at pH 2.5 relative to pH 7.

### Extraction and quantification of 2′,3′-cNMPs.

For 2′,3′-cNMP extraction, cells were harvested by centrifugation and subjected to a previously published extraction protocol (24). Briefly, frozen cell pellets were suspended in 500 μl of ice-cold acetonitrile/methanol/water (2/2/1 [vol/vol/vol]), lysed by sonication on ice, and centrifuged at 4°C at 3,000 × *g* for 10 min. The supernatant was then removed and dried to remove solvent using a vacuum centrifuge. The dry extracted material was resuspended in 250 μl of sodium phosphate buffer (50 mM [pH 7.4]) containing 0.5 μM 8-Br 3′,5′-cAMP as an internal standard (IS). The extracts were centrifuged at 12,000 × *g* for 30 min at 4°C and transferred to an LC-MS autosampler vial.

2′,3′-cNMPs were quantified via an LC-MS/MS method using 8-Br 3′,5′-cAMP as the internal standard, as previously described (20, 24). 2′,3′-cNMP concentrations were determined using a calibration curve for each analyte, and the resulting 2′,3′-cNMP levels were adjusted based on the recovery efficiency of each 2′,3′-cNMP, followed by normalization to cell number, CFU, or cell density (24). The concentration of IS was 0.5 μM in all samples for calibration, and the concentrations of 2′,3′-cNMP standards ranged from 0.02 to 20 μM. All nucleotide concentrations in stock solutions were determined via UV-visible (UV-Vis) spectrophotometry (Cary Series, Agilent Technology, Santa Clara, CA).

### LC-MS/MS conditions.

The LC-MS/MS methodology was performed as previously described (20). Briefly, a Thermo Electron linear trap quadropole-Fourier transform mass spectrometry (LTQ-FTMS) instrument was employed for sample analysis and chromatographic analysis was performed using a Shimadzu autosampler and a Dionex Ultimate 3000 dual gradient pump. LC-MS instrumentation was controlled by Xcalibur and DCMSlink software (Thermo Scientific). Samples were separated using a reversed-phase Leapsil C_18_ column (2.7 μm × 150 × 2.1 mm; Dikma Technologies, Inc., Lake Forest, CA). The mobile phase consisted of water with 0.1% formic acid (A) and methanol with 0.1% formic acid (B). The flow rate was 0.3 ml/min, and the following chromatography program was employed: 0% B from 0 to 4 min, then a gradient from 0 to 1.5% B from 4 to 15 min, followed by a gradient from 1.5% to 8% B over 15 to 20 min, followed by holding at 8% B from 20 to 25 min, then a gradient from 8% to 15% B from 25 to 28 min, followed by holding at 15% B from 28 to 35 min, and finally a gradient back to 0% B from 35 to 35.1 min prior to the column being reequilibrated by holding at 0% B from 35.1 to 45 min. The column was washed after analysis of every 2 to 4 extracts using a gradient from 0% to 100% B from 0 to 2 min, followed by holding at 100% from 2 to 10 min, then a gradient from 0% to 100% C (acetonitrile) from 10 to 12 min, followed by holding at 100% C from 12 to 20 min, followed by a final gradient from 0% to 100% A over 20 to 25 min and reequilibration at 100% A from 25 to 40 min. Electrospray ionization was performed in positive ion mode in the LTQ-FTMS instrument using a capillary voltage of 35 V, a 5-kV needle voltage, a capillary temperature of 275°C, and a 110-V tube lens voltage. Samples were detected in the ion trap using a 1-amu isolation window and a normalized collision energy of 35 eV. An activation Q of 0.250 was used, with an activation time of 30 ms. Nucleotides were detected based on the protonated parent ions and quantified using the protonated nucleobase fragment ions; peaks were integrated using Xcalibur software (Thermo Fisher).

### Growth conditions for quantification of 2′,3′-cNMPs in *S.* Typhimurium.

To quantify 2′,3′-cNMPs at different phases of growth, single colonies of the 14028s WT were inoculated into 5 ml M9-glucose medium plus 0.2% Casamino Acids and grown at 37°C overnight with shaking. Cells were subcultured to an OD_600_ of 0.05 in 50 ml fresh M9-glucose plus 0.2% Casamino Acids in 250-ml culture flasks. Cultures were grown at 37°C with aeration; 2-ml samples were collected at the mid-exponential phase (OD_600_ = 0.5), the late exponential/early stationary phase (OD_600_ = 0.9), and the late stationary phase (24 h after subculture). Cells were pelleted by centrifugation at 8,000 × *g* for 3 min. After decanting the supernatant, pellets were flash frozen in a dry ice and ethanol bath and stored at −80°C. An additional 1 ml of sample was collected, washed in 1× phosphate-buffered saline (PBS), and serially diluted. Dilutions were plated in triplicate on LB plates. Plates were incubated at 37°C overnight and were used to enumerate the CFU per milliliter present in each sample.

For 2′,3′-cNMP quantification during expression of CNPase or CNPase-inact, samples from the RNA-seq preparations (described below) were serially diluted, and CFU per milliliter were enumerated by plating dilutions on LB agar plates. Aliquots (5 ml) of the remaining samples were pelleted by centrifugation in a swinging bucket centrifuge at 3500 × *g* for 20 min. Pellets were resuspended in 1 ml PBS, transferred to a microcentrifuge tube, and pelleted by centrifugation at 8,000 × *g* for 3 min. Pellets were flash frozen and stored at −80°C prior to extraction and quantification of 2′,3′-cNMPs.

For 2′,3′-cNMP quantification upon treatment with mitomycin C (MMC), single colonies of Δ*rna* 14028s or WT 14028s, with and without pBAD33-CNP/pBAD33-CNP-inact, were inoculated into 5 ml LB medium and grown at 37°C overnight with shaking. Cells were subcultured (1:100) in 25 ml fresh LB in 250-ml culture flasks. Cultures were grown at 37°C with shaking to an OD_600_ of 0.1, at which point plasmids were induced with the addition of 0.02% l-arabinose. Cultures were then grown to an OD_600_ of 0.3 to 0.4 Each culture was split into two 10-ml cultures in 50-ml polypropylene conical tubes; one culture remained an untreated control, and the other was treated with 3 μM MMC (Sigma-Aldrich, St. Louis, MO). Tubes were returned the incubator for 90 min of treatment.

### XylE reporter assay for RNA repair operon expression.

To measure activation of expression from the *S.* Typhimurium RNA repair operon promoter, XylE reporter strains were utilized as previously described (45). JEK17 (14028s Δ*rsr*::*xylE*) was freshly transformed with pKT-CNP or pKT-CNP-inact prior to each assay. Transformants were recovered in 1 ml SOC medium at 30°C for 1 h; the entirety of each transformation was then subcultured in 10 ml LB plus carbenicillin and grown at 30°C overnight, alongside JEK17 and JEK29 (14028s Δ*rna* Δ*rsr*::*xylE*). Overnight growth was subcultured (1:50) into 25 ml LB (supplemented with carbenicillin as required to maintain plasmids) in a 250-ml culture flask. Cultures were grown at 37°C with shaking to an OD_600_ of 0.1, at which point 25 ng/ml anhydrotetracycline was added. Cultures were returned to the incubator until they reached the mid-log phase (OD_600_ = 0.3 to 0.4). Each culture was split into two 10-ml cultures in 50-ml polypropylene conical tubes; one of each pair was an untreated control, and the other was treated with 3 μM MMC. Cultures were returned to the incubator for 90 min.

At the conclusion of the treatment time, 2 ml of culture was harvested for XylE activity assays. Assays were performed as described by Kurasz et al. (45). Briefly, cells were washed and resuspended in 50 mM phosphate buffer (pH 7.4). Samples were diluted in phosphate buffer to an OD_600_of 1.0. The XylE reaction was initiated by adding 100 μl of sample to 900 μl of catechol solution (10 mM catechol [Sigma-Aldrich] and 50 mM phosphate buffer), and the conversion of catechol to 2-hydroxymuconic semialdehyde was monitored continuously at room temperature with readings taken every 10 s for 1.5 min using a Thermo Scientific Genesys 20 spectrophotometer at a wavelength (λ) of 375 nm. Each individual culture was assayed for 2 or 3 technical replicates. XylE activity was calculated as nanomoles per minute per 10^8^ cells (as previously determined by plating assays) during linear activity, based on the Beer-Lambert law and using an extinction coefficient of 44,000 L · mol^−1^ · cm^−1^ for 2-hydroxymuconic semialdehyde. The simplified equation is as follows: XylE activity = [1,000 × (ΔOD_375_/min)]/(44 × number of cells × 10^8^)].

To assay RNA repair operon expression in the presence of the cell-permeable 2′,3′-cAMP analog, Bt-cAMP, JEK12 (14028s Δ*rsr*::*xylE*-*kan*) was utilized in XylE assays as described above with the following modifications. Cultures were diluted from overnight growth to an OD_600_ of 0.05 in 50 ml LB in 500-ml culture flasks. Flask cultures were grown to an OD_600_ of 0.1 (∼1 h) and were then split into four 10-ml cultures in 50-ml conical tubes. The four tubes in each set were treated with the addition of 0.2 mM 2′,3′-cAMP, 0.2 mM Bt-cAMP, 0.2 mM sodium butyrate, or an equivalent volume of double-distilled water (ddH_2_O), respectively. Cultures were returned to the incubator, and samples were collected at 1.5-, 3-, 6-, 10-, 15-, and 25-h time points. Samples were processed and assayed for as described above (“XylE reporter assay for RNA repair operon expression”).

### qRT-PCR for RNA repair operon activity.

14028s colonies with or without pBAD33-CNP/pBAD33-CNP-inact were inoculated in 5 ml LB supplemented with chloramphenicol (35 μg/ml) and grown overnight, alongside a WT control that did not carry a plasmid. Samples were subcultured (1:100) in 25 ml LB (supplemented with chloramphenicol as required to maintain plasmids) and grown to an OD_600_ of 0.1, at which point plasmids were induced with the addition of 0.02% l-arabinose. Cultures were grown and then treated with MMC as described above (“XylE reporter assay for RNA repair operon expression”).

After the 90-min incubation, 1 ml of sample was collected for qRT-PCR. Total RNA was isolated as previously described (45). Reverse transcription and quantitative PCR were carried out simultaneously using the Bio-Rad iTaq Universal SYBR green one-step kit on a CFX Connect instrument. An equal amount of RNA was used in each reaction. Negative controls were prepared by omitting DNA/RNA template from the reaction. Standard curves were generated using 10-fold serial dilutions (to 1:10,000) of 14028s genomic DNA digested with EcoRI-HF (NEB). The starting quantity of transcript was determined by the Bio-Rad CFX software relative to the defined quantity of the standards. Data for each biological replicate is the mean of three technical replicates. To compare relative expression of the RNA repair operon, *rtcB* transcript levels (Table S2, oligonucleotides 23 and 24) were divided by *rpoD* transcript levels (Table S2, oligonucleotides 25 and 26), and the resulting values for *rtcB* levels divided by *rpoD* levels were compared between different strains and growth conditions.

### Heat shock survival assays.

To determine various strains’ abilities to survive and recover from heat shock, a heat shock survival assay was adapted from Hews et al. (83). Cultures were grown overnight in 5 ml LB (supplemented with carbenicillin as required to maintain plasmids) at 30°C. Cells were subcultured (1:100) in 15 ml LB in 50-ml polypropylene conical tubes and grown at 30°C with shaking. Upon reaching an OD_600_ of 0.1, cultures were treated with 25 ng/ml anhydrotetracycline to induce protein expression from the pKT plasmids. Cells were returned to the incubator and grown to an OD_600_ of 0.5. Cultures were then diluted 1:1 in LB medium that was prewarmed to 30°C (2 ml) or 50°C (4 ml). Samples were taken and serial diluted from 30°C *T*_0_ tubes to determine starting CFU per milliliter. Cultures were placed in a 30°C or 50°C water bath for 4 h with mild agitation every 15 min. Samples were then taken, serially diluted, and spot plated (10 μl) on LB to determine CFU per milliliter of the *T*_4 h_ cultures.

### Hydrogen peroxide survival assays.

This protocol was adapted from van der Heijden et al. (84). Cultures were grown overnight in 5 ml LB (supplemented with carbenicillin as required to maintain plasmids) at 30°C. Cells were subcultured (1:100) in 15 ml LB in 50 ml polypropylene conical tubes and grown at 30°C with shaking. Upon reaching an OD_600_ of 0.1, cultures were treated with 25 ng/ml anhydrotetracycline as an inducer of the pKT plasmids. Cells were returned to the incubator and grown to an OD_600_ of 0.5. At this point, 4 ml was removed from each culture into 15-ml conical tubes and centrifuged in a Beckman swinging bucket rotor centrifuge (3,750 × *g* for 15 min). The supernatant was decanted and the pellets were washed with 0.9% saline (wt/vol), prior to being resuspended in 1 ml 0.9% saline (OD_600_ = 1.0). Each resuspended culture was aliquoted (100 μl) into three 1.6-ml microcentrifuge tubes; 100 μl of 0.9% saline with various concentrations of H_2_O_2_ were added, such that each culture was ultimately treated with [H_2_O_2_] = 0 mM, 3.5 mM, and 7 mM. Tubes were incubated at 30°C for 2 h; samples were then taken, serially diluted, and spot plated (10 μl) on LB to determine CFU/ml of each culture.

### Treatment with 5′-*O*-butyryl 2′,3′-cGMP.

Cultures of E. coli WT and Δ*rna* were grown in 250-ml baffled Erlenmeyer flasks containing 30 ml of M9 medium (0.4% glucose and 0.2% Casamino Acids) containing appropriate antibiotics and were grown at 37°C with shaking. An initial optical density time point was taken after cells had grown to an OD_600_ of ∼0.6; at this point cells were treated with 500 μM Bt-cGMP and allowed to grow for an additional 24 h. Time points were taken after 90 min, 6 h, and 24 h.

### Occurrence of 2′,3′-cNMPs in other organisms.

Publicly available metabolite databases were searched for the occurrence of 2′,3′-cNMPs. These databases included the Human Metabolome Database (HMDB; hmdb.ca), Metabolomics Workbench (MW; metabolomicsworkbench.org), MetaboLights and CheBI (EMBL-EBI; ebi.ac.uk), and the Biological Magnetic Resonance Database (brmb.wisc.edu). The databases were searched using either the structure of the neutral and deprotonated (−1) forms of each 2′,3′-cNMP, the molecular formulas for the neutral and deprotonated (−1) 2′,3′-cNMPs, or the following search terms: 2′,3′-cyclic, 2′,3′-cyclic AMP, 2′,3′-cyclic CMP, 2′,3′-cyclic UMP, 2′,3′-cyclic GMP, adenosine 2′,3′-cyclic phosphate, guanosine 2′,3′-cyclic phosphate, cytidine 2′,3′-cyclic phosphate, and uridine 2′,3′-cyclic phosphate. Results, including data set reference numbers, are listed in Table 2.

### Data availability.

Gene expression data for the WT and Δ*rna* strains have been submitted to ArrayExpress at EMBL-EBI (http://www.ebi.ac.uk/arrayexpress/) under accession number E-MTAB-6095. Gene expression data for Escherichia coli WT pKT-CNP and pKT-CNP-inact have been deposited in the NCBI Gene Expression Omnibus (GEO) database under accession number GSE114871. Gene expression data for *S.* Typhimurium 14028s pKT-CNP and pKT-CNP-inact have been deposited to the NCBI GEO database under accession number GSE173726. Gene expression data for E. coli Δ*rna* strains expressing pKT-CNP and pKT-CNP-inact have been deposited to the NCBI GEO database under accession number GSE182184. Gene expression data for *S.* Typhimurium 14028s Δ*rna* strains expressing pKT-CNP and pKT-CNP-inact have been deposited to the NCBI GEO database under accession number GSE184190. Gene expression data for the *S.* Typhimurium 14028s Δ*rna* strain compared to the WT strain have been deposited to the NCBI GEO database under accession number GSE184189.
