# Supplementary material for: Cellular Effects of 2′,3′-Cyclic Nucleotide Monophosphates in Gram-Negative Bacteria
Source: J Bacteriol. 2022 Jan 18;204(1):e00208-21. doi: 10.1128/JB.00208-21 (PMC8765455; doi:10.1128/JB.00208-21)
Supplement: Supplemental file 6 — Supplemental text, Tables S1 and S2, and Fig. S1 to S8. Download JB.00208-21-s0001.pdf, PDF file, 3.1 MB [file jb.00208-21-s0001.pdf]

Cellular Effects of 2´,3´-Cyclic Nucleotide Monophosphates in Gram-Negative Bacteria

Yashasvika Duggal, Jennifer E. Kurasz, Benjamin M. Fontaine, Nick J. Marotta, Shikha S. Chauhan, Anna C. Karls, and Emily E. Weinert

SUPPLEMENTAL METHODS, TABLES, AND FIGURES

Supplemental Methods

General conditions for chemical synthesis and characterization. All reactions were conducted in flame-dried glassware under a nitrogen atmosphere using anhydrous solvents (Drisolv®, MilliporeSigma) and magnetic stirring. For thin layer chromatography (TLC), alumina-backed silica gel 60 F_{254} plates were used with a mobile phase of 6/1.5/1.5/1.5/0.25 EtOAc/MeCN/MeOH/H_2O/NH_4OH; the plates were visualized under UV light. Diethylaminoethyl (DEAE) Sephadex® A-25 resin (GE Healthcare) was utilized for anion-exchange chromatography. ^{1}H, ^{13}C, and ^{31}P nuclear magnetic resonance (NMR) spectra were recorded on Varian INOVA 400 and Mercury 300 spectrometers. Chemical shifts are reported in ppm and are referenced to the residual solvent signal for ^{1}H and ^{13}C NMR (for ^{1}H NMR: DMSO-d6 = 2.50 ppm; for ^{13}C NMR: DMSO-d6 = 39.52 ppm). ^{31}P NMR chemical shifts are referenced externally to the phosphoric acid signal (0 ppm) in DMSO-d6. *J*-coupling values are reported in hertz (Hz). A Thermo LTQ FTMS was used to collect high-resolution mass spectra (HRMS). NMR spectra for all compounds are provided below.

Preparation of DEAE Sephadex® A-25 bicarbonate form and compound purification. DEAE Sephadex® A-25 resin was hydrated in 100 mM NH_4HCO_3, poured into a flash chromatography column, and washed with several column volumes of 100 mM NH_4HCO_3, followed by several column volumes of water. The crude material was dissolved in 5-10 mL of water, and the pH was adjusted to ~7-8. This solution was loaded onto the column and eluted with water for several fractions. Subsequently, the ionic strength of the eluent was slowly increased by gradual addition of aqueous NH_4HCO_3 to elute the desired 2´,3´-cNMP

analogs as the ammonium salts. The product-containing fractions were concentrated to dryness by lyophilization to remove NH_4HCO_3.

Conversion of 3’-UMP (disodium salt) to 3’-UMP (mixed pyridinium/sodium salt). To enhance solubility in pyridine, the disodium salt of 3’-UMP was converted to the mixed pyridinium/sodium salt by passing the nucleotide over a column of Amberlite® IR-120 (pyridinium form). To this end, the cation-exchange resin (free acid form) was hydrated in 10% (v/v) aqueous pyridine, and washed with several column volumes of 10% (v/v) aqueous pyridine, followed by washing with several column volumes of water. 3’-UMP (disodium) was dissolved in water and slowly passed through the column. The UV-active fractions were lyophilized to obtain 3’-UMP (mixed pyridinium/sodium salt). The presence of the pyridinium cation was confirmed by ^{1}H NMR analysis.

Conversion of 3’-GMP (disodium salt) to 3’-GMP (mixed pyridinium/sodium salt). The similar procedure was followed to convert the disodium salt of 3’-GMP was converted to the mixed pyridinium/sodium salt as in described above for the conversion of disodium salt of 3’-UMP to the mixed pyridinium/sodium salt of 3’-UMP.

Synthesis of 5’-*O*-butyryl adenosine 2′,3′-cyclic monophosphate (Bt-cAMP). To a vigorously stirred solution of 3’-AMP (free acid) (0.144 mmol) and *N*, *N*’-dicyclohexyl-4-morpholinecarboxamidine (0.136 mmol) in pyridine (6 mL), butyric anhydride (1.15 mmol) was added dropwise. The reaction was stirred at room temperature (RT) and monitored by silica gel TLC. Additional butyric anhydride was added as necessary to drive the reaction forward. Upon completion of the reaction, the solution was cooled in an ice-water bath and quenched by dropwise addition of MeOH (5 mL). After stirring for 3 h, the reaction was diluted with toluene and concentrated *in vacuo*. The residue was subjected to azeotropic distillation with several additional portions of toluene, subsequently partitioned between H_2O and Et_2O, and washed with several portions of Et_2O and CH_2Cl_2. The aqueous phase was concentrated *in vacuo* by azeotropic distillation with EtOH and the crude material was purified by anion-exchange chromatography over DEAE-Sephadex® A-25 resin, as detailed above. The compound was obtained in 59% yield as the ammonium salt

(35.6 mg). **^{1}H NMR** (400 MHz, DMSO-d6) δ 8.39 (s, 1H), 8.18 (s, 1H), 7.56 (s, br, 2H), 7.30 (t, br, J = 47.0 Hz, 4H), 6.18 (d, J = 3.7 Hz, 1H), 5.34 (ddd, J = 9.2, 7.1, 3.7 Hz, 1H), 4.87 (ddd, J = 11.5, 7.0, 4.9 Hz, 1H), 4.41 – 4.27 (m, 2H), 4.24 – 4.11 (m, 1H), 2.25 (td, J = 7.3, 2.0 Hz, 2H), 1.48 (q, J = 7.4 Hz, 2H), 0.82 (t, J = 7.4 Hz, 3H). **^{13}C NMR** (100 MHz, DMSO-d6) δ 172.60, 155.68, 152.30, 149.00, 140.10, 119.00, 88.23, 82.06, 78.49, 75.85, 63.20, 35.12, 17.86, 13.42. **^{31}P NMR** (162 MHz, DMSO-d6) δ 16.54. **HRMS** (ESI-, *m/z*) [M-H]$^{-}$ calculated for $C_{14}H_{17}N_5O_7P^{-}$ 398.0871, found 398.0871.

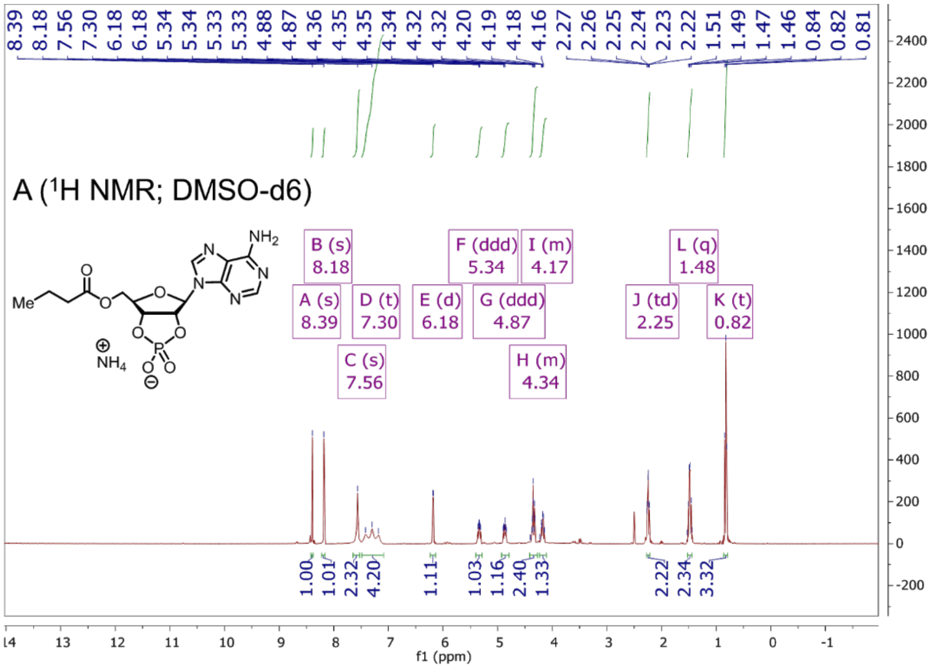

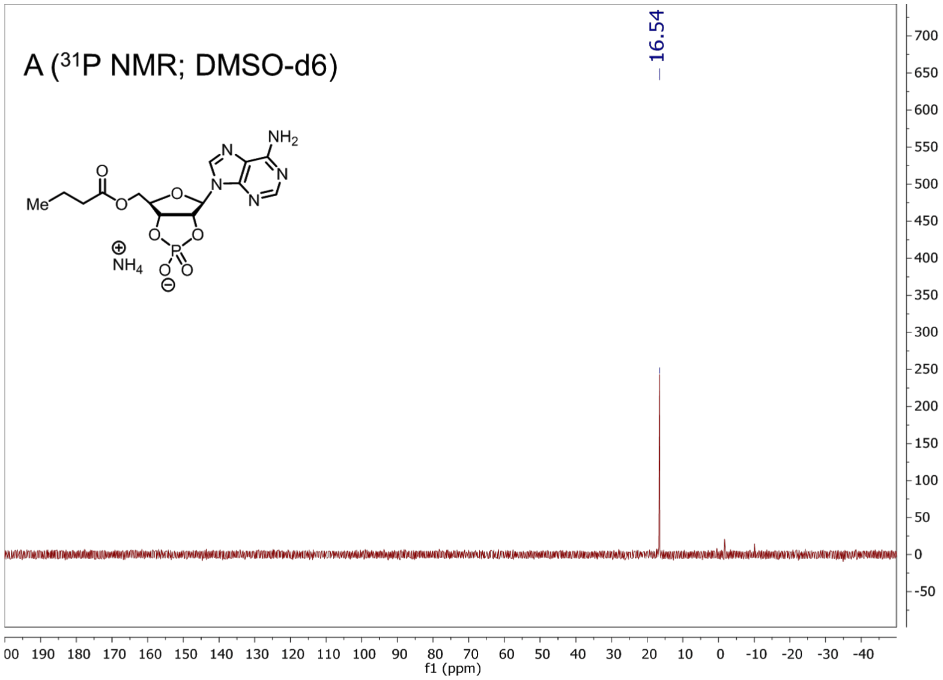

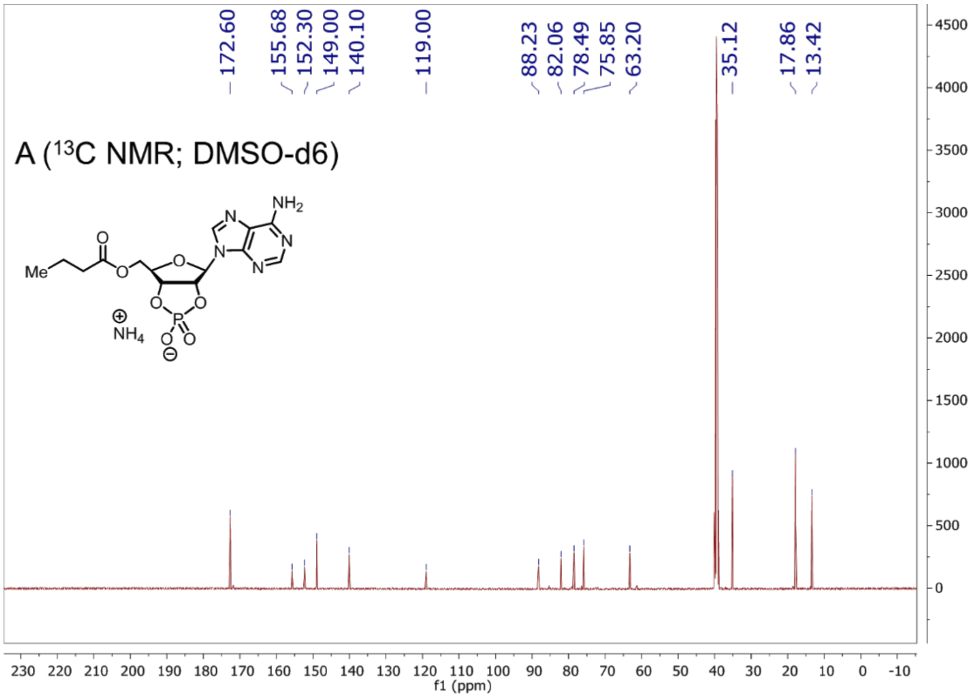

Synthesis of 5’-*O*-butyryl uridine 2′,3′-cyclic monophosphate (Bt-cUMP). The compound was synthesized from 3’-UMP (mixed pyridinium/sodium salt) in analogy to the preparation of Bt-cAMP. The compound was obtained in 72% yield as the ammonium salt (69.5 mg). **^{1}H NMR** (400 MHz, DMSO-d6) δ

11.44 (s, br, 1H), 7.69 (d, *J* = 8.0 Hz, 1H), 7.25 (s, br, 4H), 5.83 (d, *J* = 3.0 Hz, 1H), 5.65 (d, *J* = 8.0 Hz, 1H), 4.76 (td, *J* = 7.6, 3.1 Hz, 1H), 4.56 (dt, *J* = 12.6, 6.3 Hz, 1H), 4.29 (dd, *J* = 11.3, 3.2 Hz, 1H), 4.26 – 4.10 (m, 2H), 2.30 (t, *J* = 7.3 Hz, 2H), 1.53 (h, *J* = 7.3 Hz, 2H), 0.87 (t, *J* = 7.4 Hz, 3H). **^{13}C NMR** (100 MHz, DMSO-d6) δ 172.58, 163.13, 150.23, 142.52, 102.08, 91.41, 81.99, 78.19, 75.32, 63.50, 35.11, 17.85, 13.42. **^{31}P NMR** (162 MHz, DMSO-d6) δ 16.24. **HRMS** (ESI-, *m/z*) [M-H]$^-$ calculated for $C_{13}H_{16}N_2O_9P^-$ 375.0599, found 375.0597.

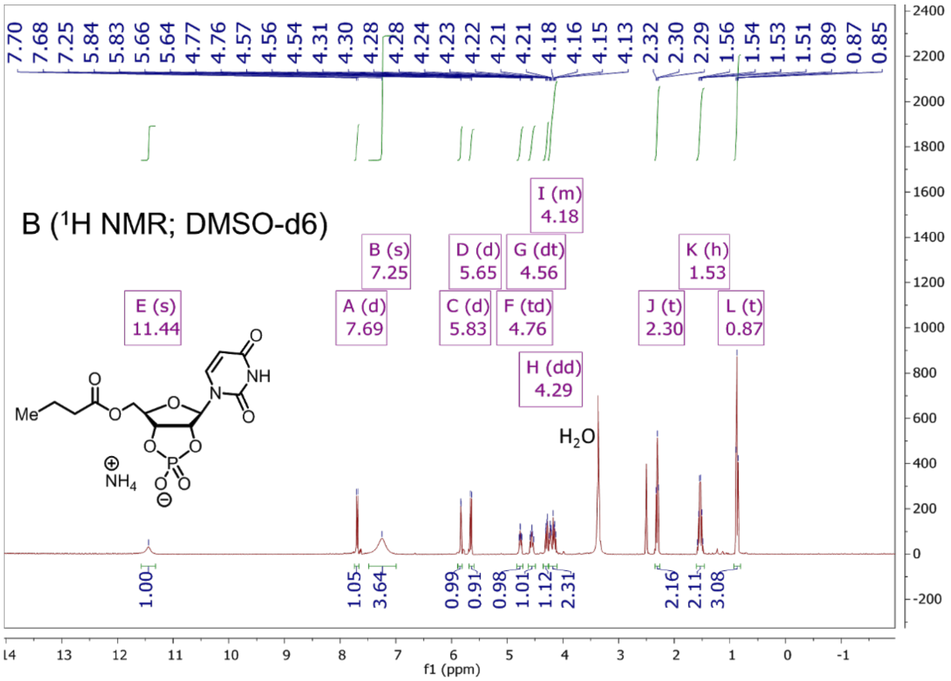

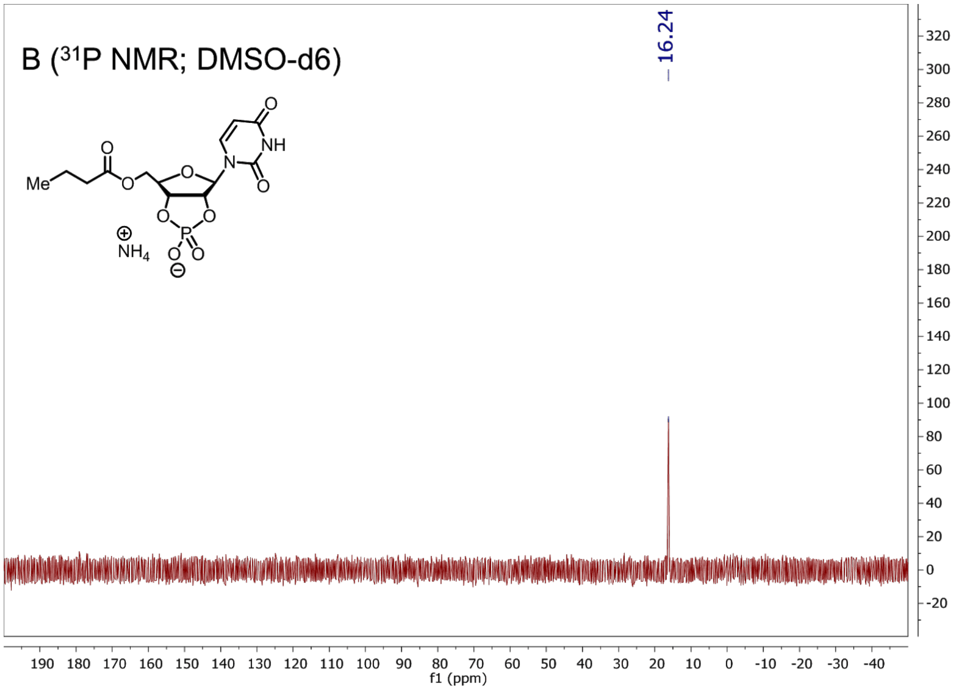

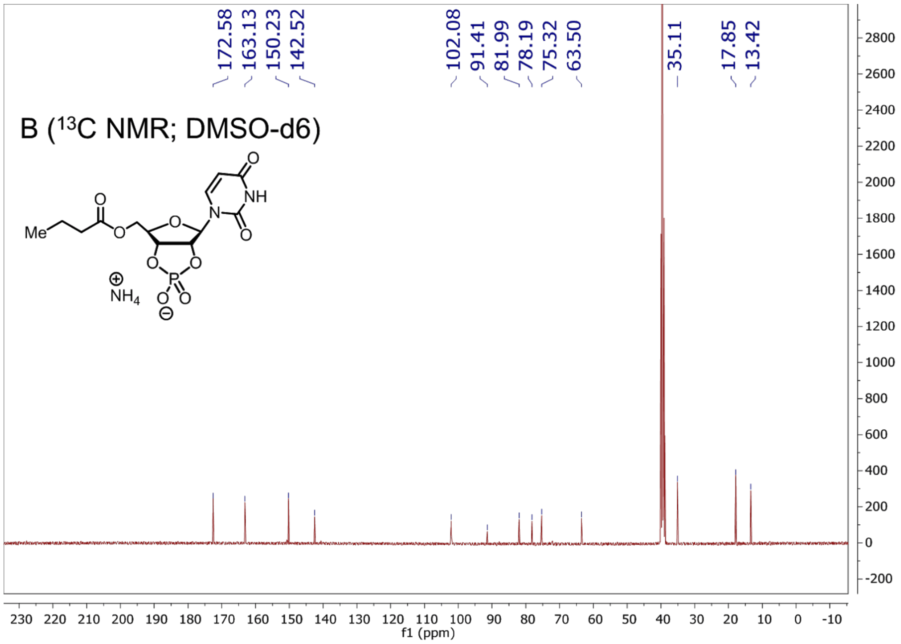

Synthesis of 5'-*O*-benzoyl uridine 2´,3´-cyclic monophosphate (Bz-cUMP). The compound was prepared by treating 3'-UMP (mixed pyridinium/sodium salt) with benzoic anhydride, in analogy to the synthesis of Bt-cUMP. The compound was obtained in 15% yield as the ammonium salt (38.4 mg). **^{1}H NMR** (400 MHz, DMSO-d6) δ 7.99 (dd, *J* = 8.2, 1.0 Hz, 2H), 7.71 (d, *J* = 8.1 Hz, 1H), 7.69 – 7.63 (m,

1H), 7.54 (t, J = 7.7 Hz, 2H), 5.87 (d, J = 3.0 Hz, 1H), 5.57 (d, J = 8.0 Hz, 1H), 4.81 (td, J = 7.6, 3.1 Hz, 1H), 4.70 (ddd, J = 13.0, 7.2, 5.6 Hz, 1H), 4.54 (dd, J = 11.6, 3.8 Hz, 1H), 4.50 – 4.34 (m, 2H). **^{13}C NMR** (100 MHz, DMSO-d6) δ 165.50, 163.13, 150.24, 142.63, 133.54, 129.31, 128.83, 102.04, 91.64, 82.02, 78.33, 75.39, 64.33. **^{31}P NMR** (162 MHz, DMSO-d6) δ 16.31. **HRMS** (ESI-, *m/z*) $[M-H]^-$ calcd for $C_{16}H_{14}N_2O_9P^-$ 409.0442, found 409.0446.

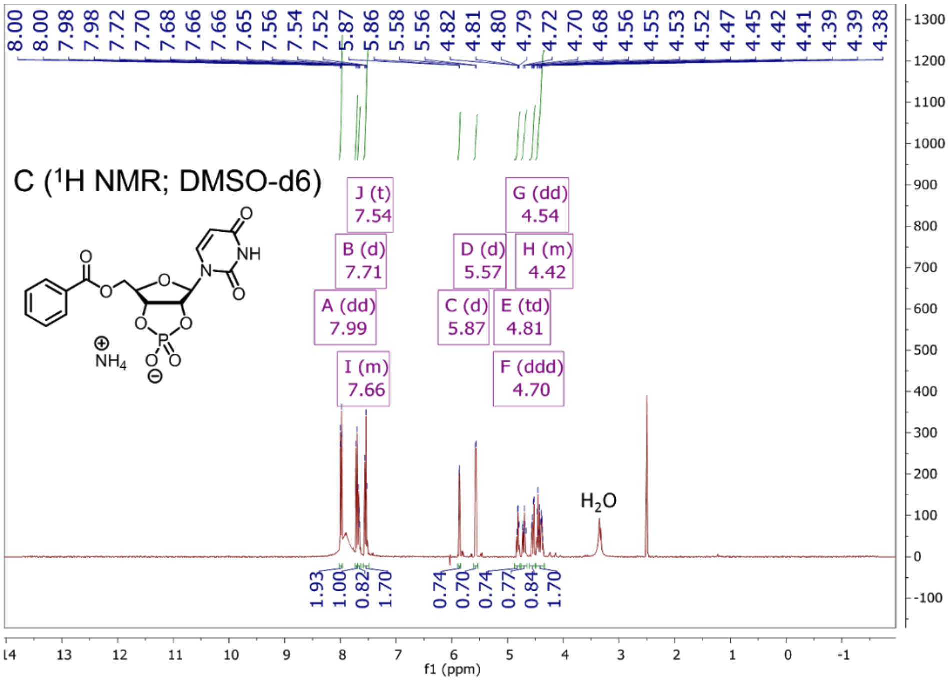

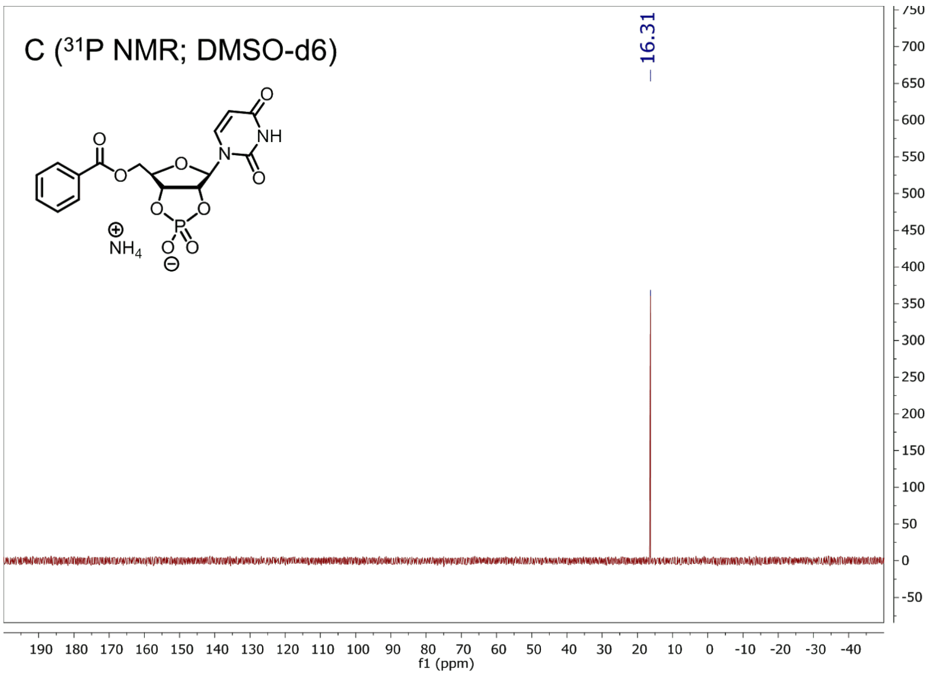

Synthesis of 5'-*O*-butyryl guanosine 2´, 3´-cyclic monophosphate (Bt-cGMP). The compound was synthesized from 3'-GMP (mixed pyridinium/sodium salt) in analogy to the preparation of Bt-cAMP. The compound was obtained in 25% yield as the ammonium salt (34.5 mg). **^{1}H NMR** (400 MHz, DMSO-d6) δ 10.72 (s, 1H), 7.91 (s, 1H), 7.28 (t, br, *J* = 50.0 Hz, 4H), 6.62 (s, br, 2H), 5.97 (d, *J* = 3.6 Hz, 1H), 5.07 (ddd, *J* = 9.2, 7.2, 3.6 Hz, 1H), 4.82 – 4.76 (m, 1H), 4.34 – 4.26 (m, 2H), 4.17 – 4.12 (m, 1H), 2.31 (t, *J* = 7.2 Hz, 2H), 1.57 – 1.48 (m, 2H), 0.88 (t, *J* = 7.6 Hz, 3H). **^{13}C NMR** (100 MHz, DMSO-d6) δ 173.06, 157.20, 154.35, 151.39, 136.29, 117.15, 88.11, 82.46, 78.95, 75.97, 63.98, 35.55, 18.29, 13.85. **^{31}P NMR** (162 MHz, DMSO-d6) δ 16.40. **HRMS** (ESI-, *m/z*) $[M-H]^-$ calculated for $C_{14}H_{21}N_8O_8P^-$ 432.1158, found 432.0928.

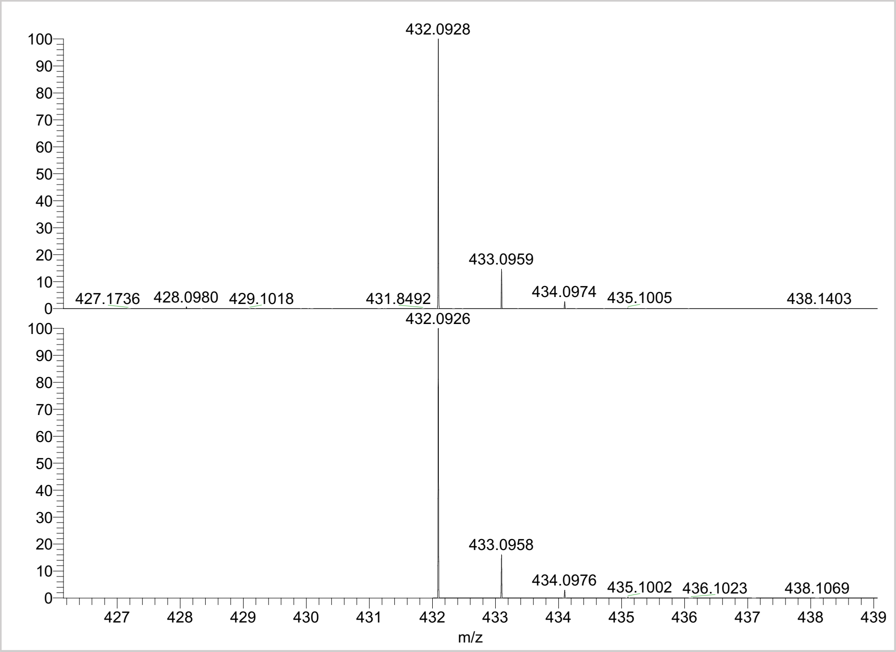

432.0928
433.0959
434.0974
427.1736
428.0980
429.1018
431.8492
435.1005
438.1403
432.0926
433.0958
434.0976
435.1002
436.1023
438.1069
m/z

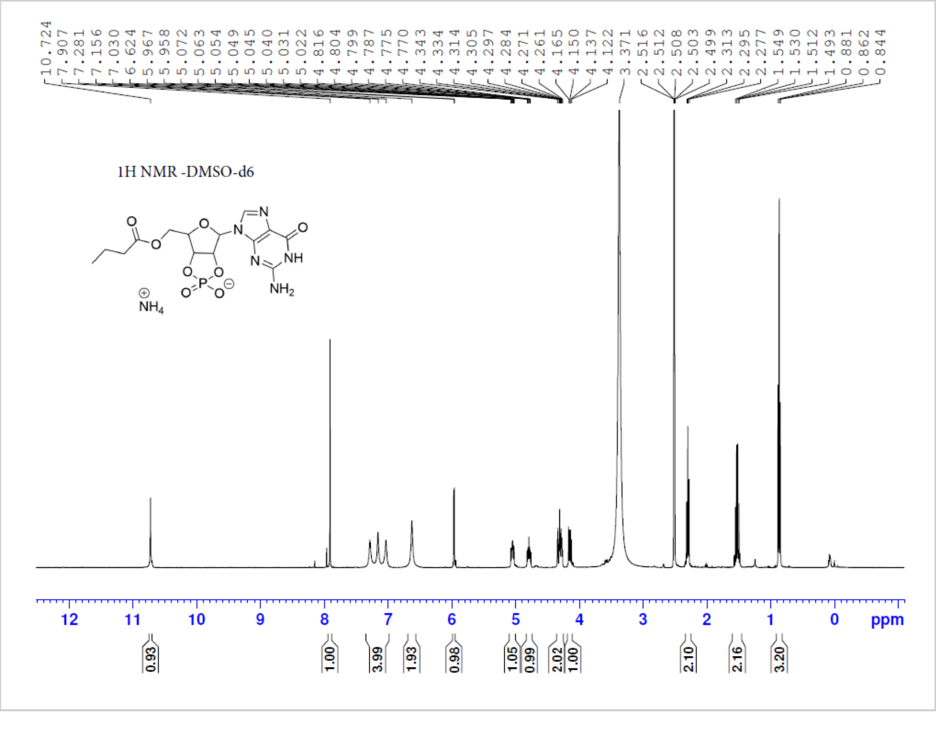

1H NMR -DMSO-d6
NH
NH2
NH4
ppm

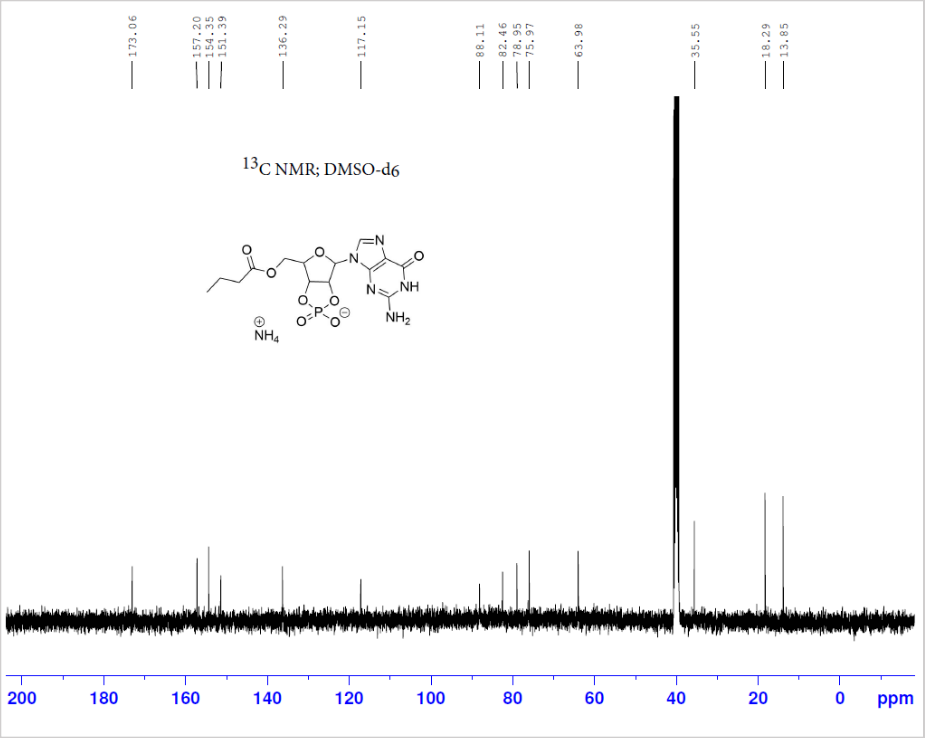
173.06
157.20
154.35
151.39
136.29
117.15
88.11
82.46
78.95
75.97
63.98
35.55
18.29
13.85
^{13}C NMR; DMSO-d6
200
180
160
140
120
100
80
60
40
20
0
ppm

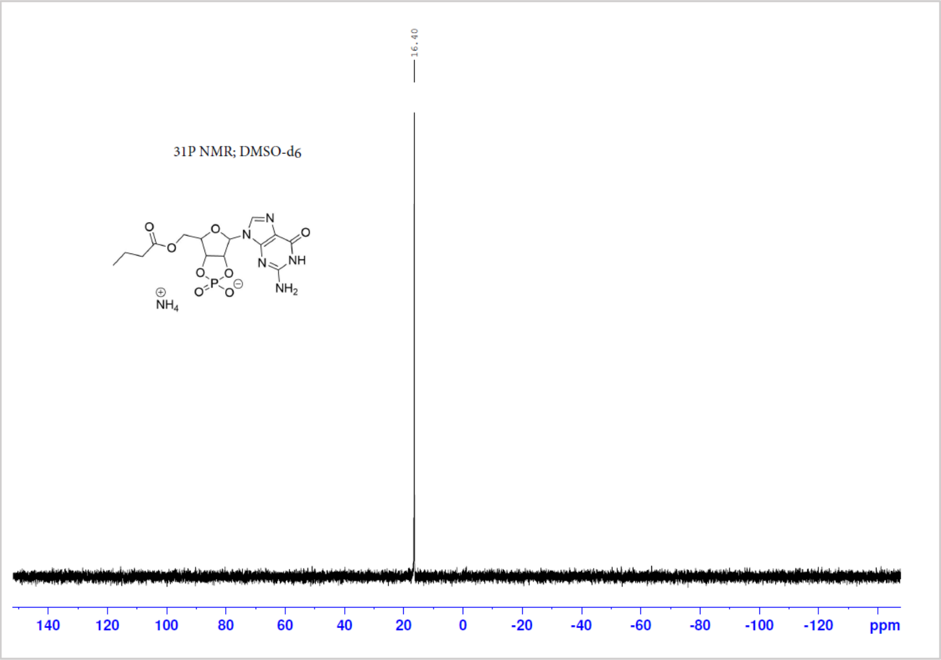
16.40
31P NMR; DMSO-d6
140
120
100
80
60
40
20
0
-20
-40
-60
-80
-100
-120
ppm

Table S1. Bacterial strains and plasmids used in this study.

Strain/Plasmid	Description or Construction	Source or reference
E. coli		
BW25113	*lacI*^{q} *rrnB*$_{T14}$ Δ*lacZ*$_{WJ16}$ *hsdR514* Δ*araBAD*$_{AH33}$ Δ*rhaBAD*$_{LD78}$	(1)
Δ*rna*	BW25113 Δ*rna*::*kan*	(1)
***S.* Typhimurium**		
ATCC 14028s	Wild-type	ATCC
JEK20	14028s Δ*rna*	This study
JEK12	14028s Δ*rsr*::*xylE-kan*	(2)
JEK17	14028s Δ*rsr*::*xylE*	(2)
JEK29	14028s Δ*rna* Δ*rsr*::*xylE*	This study
JEK30	14028s Δ*recA*	This study
SGD_yjbE_Kan	14028s Δ*yjbE*::*kan*	BEI Resources, ATCC
SGD_wcaI_Kan	14028s Δ*wcaI*::*kan*	BEI Resources, ATCC
SGD_csgD_Kan	14028s Δ*csgD*::*kan*	BEI Resources, ATCC
**Plasmids**		
pKT-*CNP*	Expresses mammalian CNPase from a Tet promoter; Amp^{R}	(3)
pKT-*CNP-inact*	Expresses catalytically inactive CNPase variant (H73L/H152L) from a Tet promoter; Amp^{R}	(3)
pKT-*CNP*(*cat*)	pKT-*CNP* with replacement of antibiotic resistance marker; Cam^{R}	This study
pKT-*CNP-inact* (*cat*)	pKT-*CNP-inact* with replacement of antibiotic resistance marker; Cam^{R}	This study
pBAD33-*CNP*	Expresses mammalian CNPase from an *araBAD* promoter; Cam^{R}	This study
pBAD33-*CNP-inact*	Expresses catalytically inactive CNPase variant (H73L/H152L) from an *araBAD* promoter; Cam^{R}	This study

Table S2. Oligonucleotides used in this study.

Number	Name	Sequence (5’→3’)
**Confirming gene deletions**		
1	*citT*-fwd	ATT AGC TGG TTG CAG TGG TTC CTC
2	*kan*^{R}-rev	GTC ATA GCC GAA TAG CCT CTC CAC
3	*kan*^{R}-fwd	TCG CAG CGC ATC GCC TTC TAT C
4	*rnk*-rev	CAC TTC GCC ATC GCT AAG ATT GC
5	ck-rna-F	AAC ATT ACG CCA ACG CAG GTT
6	ck-rna-R	GTT CAT GGT GAC GAC ATC GTT
7	ck-recA-F	CGG TTC AAT ACC AAG TTG CAT GAC
8	ck-recA-R	GAA TGG CGG CTT CGT TTT GC
9	ck-yjbE-F	TGT TTT GGT TGT GTA TAC TCG GTA GC
10	ck-yjbE-F	ACC ACA CCG GAG TGT GGT TAT GG
11	ck-wcaI-F	TTT GCA GCA TGG TGA GAT CGC
12	ck-wcaI-R	AGC ATG GCA GTT ACC GCT GG
13	ck-csgD-F	TCC TAT CGA AGA GAC GGT CTG GTC G
14	ck-csgD-R	TGC TGT CCA GGT TAA TGC CAC G
**Site-directed mutagenesis**		
15	*CNP*-H73L-fwd	GGG TGT TCT GCT GTG CAC CAC GAA ATT C
16	*CNP*-H73L-rev	GAA TTT CGT GGT GCA CAG CAG AAC ACC C
17	*CNP*-H152L-fwd	GTG CGC TGG TTA CCC TGG GTT GTG
18	*CNP*-H152L-rev	CAC AAC CCA GGG TAA CCA GCG CAC
**PIPE cloning for replacement of *bla* with *cat***		
19	pKT vector rev	ACT CTT CCT TTT TCA ATA TTA TTG AAG CAT
20	pKT vector fwd	CTG TCA GAC CAA GTT TAC TCA TAT ATA C
21	cam^R insert fwd	ATG CTT CAA TAA TAT TGA AAA AGG AAG AGT ATG GAG AAA AAA ATC ACT GGA TAT ACC
22	cam^R insert rev	GTA TAT ATG AGT AAA CTT GGT CTG ACA G TTA CGC CCC GCC CTG
**qRT-PCR**		

23	rtcB Q2 F	GTG ATG AGC CGA ACG AAA GC
24	rtcB Q2 R	CCG CAT CAA TAT CTT TAT ACG CC
25	rpoD-RT F	AAC GAA TAA GTG TGG ATA CCG
26	rpoD-RT R	TCT TCC ATT ACC TGA ATA CCC

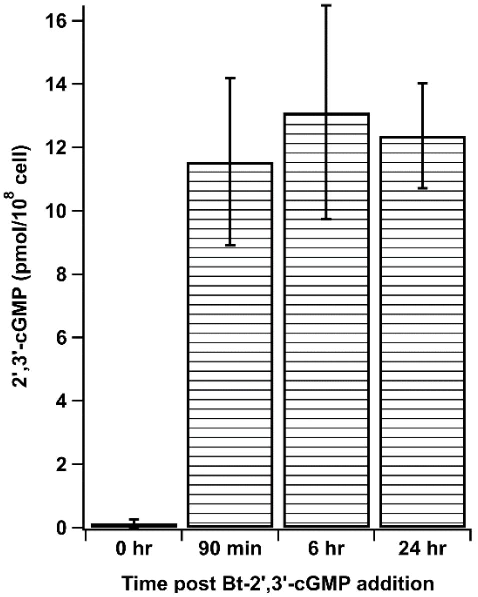

Figure S1. Treatment with 5’-O-butyryl 2’,3’-cGMP results in 2’,3’-cGMP production in *E. coli*. To assess whether 5’-O-butyryl 2’,3’-cGMP (Bt-2’,3’-cGMP) could enter the cell and be hydrolyzed to yield 2’,3’-cGMP, *E. coli* Δ*rna* cells were grown at 37°C in M9 media supplemented with 0.4% glucose and 0.2% casamino acids to mid-log phase (OD_{600} ~ 0.6) and then treated with 500 μM Bt-cGMP. The 0 hr sample was taken immediately prior to addition of Bt-cGMP. Samples were taken at 90 min, 6 hr, and 24 hr after Bt-2’,3’-cGMP addition; 2’,3’-cGMP levels were quantified by LC-MS/MS, as described in Materials and Methods. Each time point is representative of 3 biological replicates and error bars represent standard deviation.

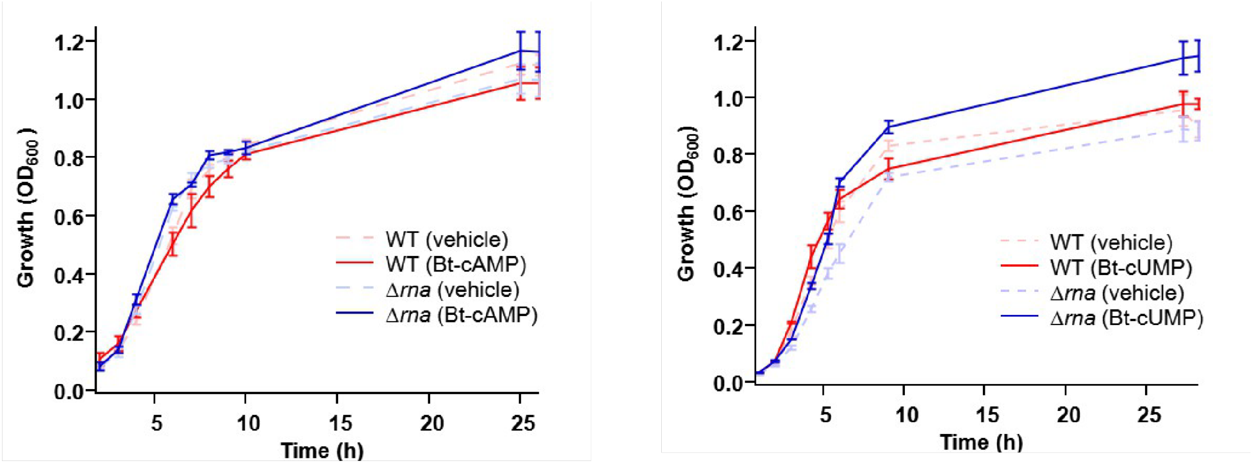

Figure S2. Treatment with 5’-O-butyryl 2’,3’-cAMP or 5’-O-butyryl 2’,3’-cUMP does not impact *E. coli* cell growth. *E. coli* WT or Δ*rna* cells were grown in M9 media supplemented with 0.4% glucose and 0.2% casamino acids. The growth medium was additionally supplemented with 1 mM of (A) 5’-O-butyryl 2’,3’-cAMP (Bt-cAMP) or (B) 5’-O-butyryl 2’,3’-cUMP (Bt-cUMP) to assess whether addition of the cell permeable 2’,3’-cNMP analogs would affect growth over time, as determined by cell density (OD_{600}). For both nucleotides, statistical analyses by Student’s t-test comparing treated vs. untreated cultures (vehicle) determined that their inclusion in growth media does not significantly impact cell growth ($p > 0.05$). Each point on the graph represents the average of 3 biological replicates.

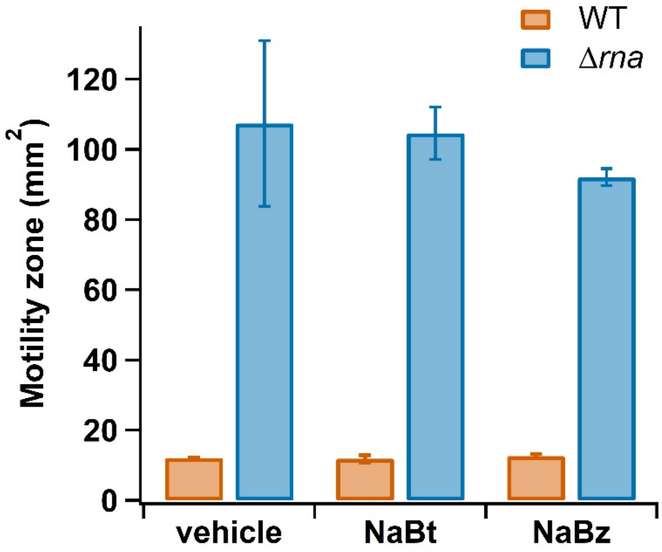

Figure S3. Addition of sodium butyrate or sodium benzoate does not impact motility in *E. coli* WT or Δ*rna*. Motility was assessed on soft agar motility plates containing 1 mM sodium butyrate (NaBt) or sodium benzoate (NaBz) in the media and compared to an untreated control (vehicle). Statistical analyses by Student's t-test to compare treated vs. untreated samples determined there is no significant difference in motility for WT or Δ*rna* strains with the addition of NaBt or NaBz ($p > 0.05$). Data is representative of 3 biological replicates and error bars represent ± 1 standard deviation.

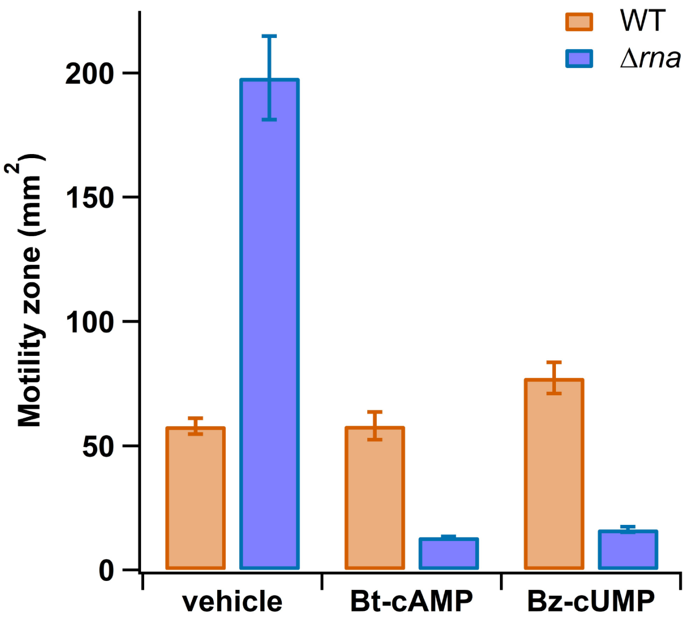

Figure S4. 5'-O-benzoyl 2',3'-cUMP inhibits the hypermotility phenotype of *E. coli* Δ*rna*. Motility was assessed on soft agar motility plates containing 1 mM Bt-cAMP, 1 mM 5'-O-benzoyl 2',3'-cUMP (Bz-cUMP) in the media and compared to an untreated control (vehicle). Statistical analyses by Student's t-test to compare motility in treated vs. untreated samples determined that Bz-cUMP inhibits the hypermotility of the Δ*rna* strain (for Δ*rna* Bz-cUMP vs. vehicle, $p < 0.05$) to a similar degree as Bt-cAMP (for Δ*rna* Bt-cAMP vs. Bz-cUMP, $p > 0.05$). Neither nucleotide analog had a significant impact on motility for the WT strain ($p > 0.05$). Motility of both strains was greater (compared to Fig. S2) due to higher humidity levels. Data is representative of 3 biological replicates and error bars represent ± 1 standard deviation.

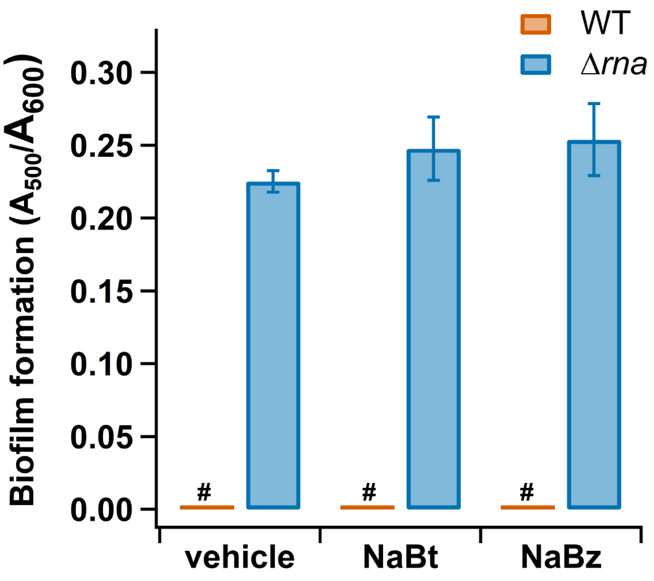

Figure S5. Addition of sodium butyrate or sodium benzoate does not impact biofilm formation in *E. coli* WT or Δ*rna*. Biofilm was assessed by Congo red staining for cells that were treated with 500 µM sodium butyrate (NaBt) or sodium benzoate (NaBz) compared to an untreated control (vehicle). Statistical analyses by Student's t-test to compare treated vs. untreated samples determined there is no significant difference in biofilm formation for either strain with the addition of NaBt or NaBz ($p > 0.05$). Data is representative of 3 biological replicates and error bars represent ± 1 standard deviation.

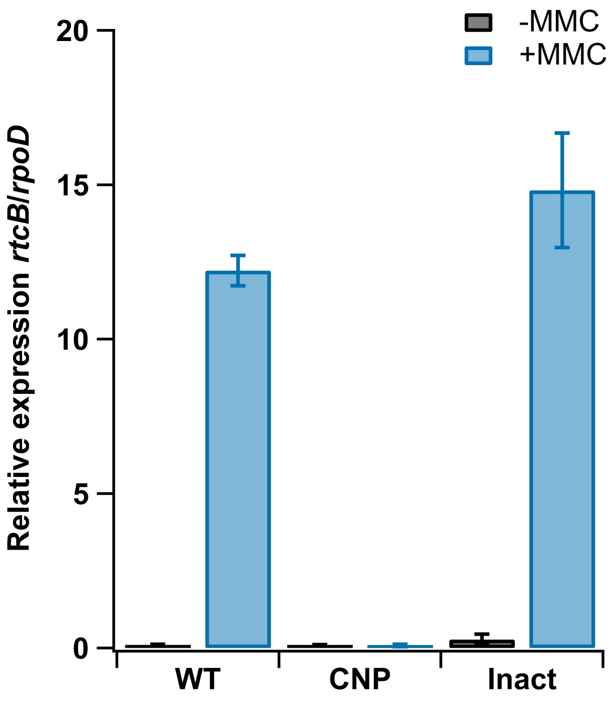

Figure S6. Quantification of *rtcB* transcripts reveals CNPase-dependent inhibition of transcription from the *S.* Typhimurium RNA repair operon promoter. *S.* Typhimurium 14028s WT, 14028s (pBAD33-*CNP*), or 14028s (pBAD-*CNP-inact*) cells were treated with 3 µM mitomycin C (MMC) for 90 min to activate transcription of the *rsr-yrlBA-rtcBA* RNA repair operon. qRT PCR was performed as described in Methods and Materials using 3 biological replicates with 3 technical replicates for each strain and condition; error bars represent ±1 standard deviation. Statistical analysis by Student's t-test indicated that cells expressing CNP-Inact produced a similar level of *rtcB* transcripts upon treatment with MMC as WT cells (for WT + MMC vs. Inact + MMC $p < 0.05$); however, cells expressing catalytically active CNPase were unable to generate *rtcB* transcripts during treatment with MMC (for WT + MMC or Inact + MMC vs. CNPase + MMC, $p < 0.05$; for CNPase + MMC vs. untreated, $p > 0.05$).

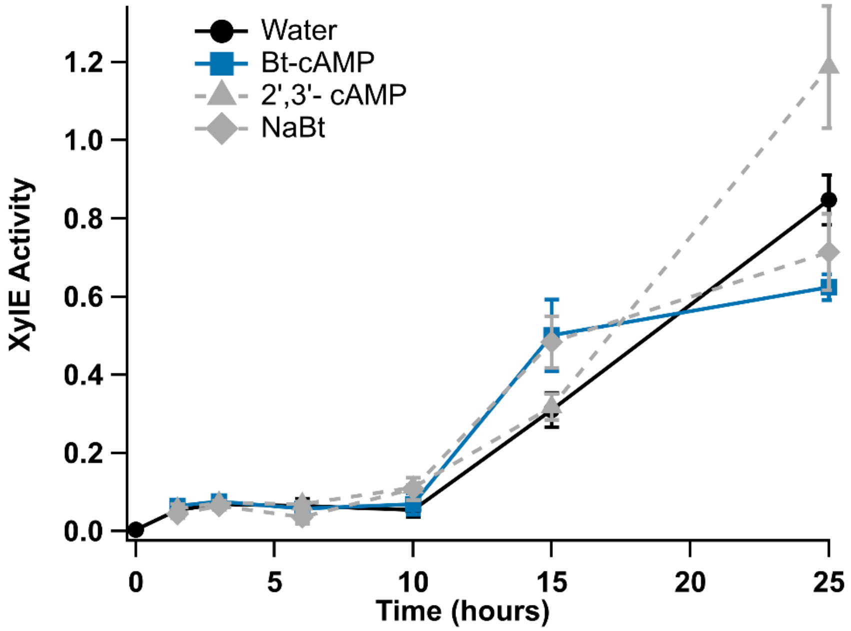

Figure S7. Effects of Bt-cAMP on RNA repair operon expression are confounded by the effects of cellular stress in stationary phase. To determine whether 2',3,'-cNMPs contribute to the activation of transcription from the RNA repair operon promoter, a XylE reporter assay was performed. Three biological replicates of the reporter strain JEK12 were treated with 0.2 mM Bt-cAMP, 0.2 mM NaBt, 0.2 mM 2',3'-cAMP (adenosine 2',3'-cyclic monophosphate sodium salt; Sigma-Aldrich, St. Louis, MO), or water (empty vehicle). Samples were collected periodically for up to 25 h and assayed for XylE activity as described in Materials and Methods. All samples, regardless of treatment, showed enhanced XylE activity after 10 h of growth with activity continuing to increase to the end of the experiment, likely due to cell stress experienced during extended growth in batch culture. Error bars represent ±1 standard deviation.

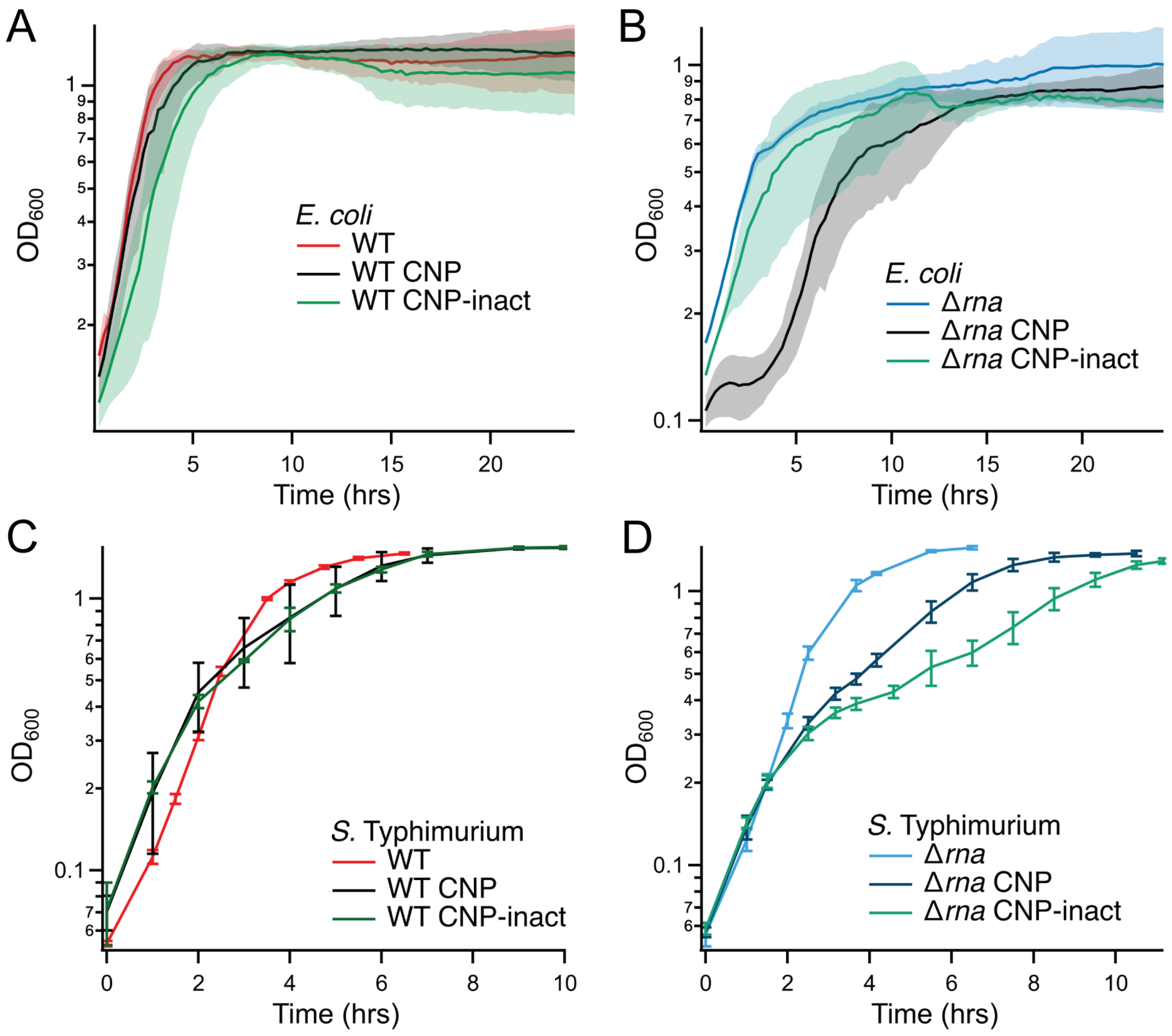

Figure S8. Effects of plasmids pKT-CNP and pKT-CNP-inact on cell growth. Growth curves were performed for *E. coli* and *S*. Typhimurium strains with and without the pKT-CNPase and pKT-CNP-Inact plasmids. Cultures of (A) *E. coli* WT (B) *E. coli Δrna* (C) *S*. Typhimurium WT and (D) *S*. Typhimurium *Δrna* strains were grown and monitored as described in the Material and Methods; OD_{600} was measured at 15 min intervals for *E. coli* and 30-60 min intervals for *S*. Typhimurium. Expression of *CNP* or *CNP-Inact* was induced with 25 ng/mL anhydrotetracycline at OD_{600} ~0. 1. Each line represents the mean for 3 or more biological replicates at each time point and shading represents ± 1 standard deviation. The calculated doubling times (in minutes) for *E. coli:* WT 53 ±6, WT+pKT-CNP 63 ±9, WT+pKT-CNP-Inact 69 ±10, *Δrna* 88 ±6, *Δrna*+pKT-CNP 103 ±25, *Δrna*+pKT-CNP-Inact 155 ±65; and for *S*. Typhimurium: WT 40 ±1, WT+pKT-CNP 85 ±5, WT+pKT-CNP 91 ±9, *Δrna* 41 ±3, *Δrna*+pKT-CNP 94 ±12, *Δrna*+pKT-CNP-Inact 99 ±3. Differences in doubling times are statistically significant by Student's t-test analysis for: *E. coli* WT vs. WT+pKT-CNP, WT vs. WT+pKT-CNP-Inact, WT vs. *Δrna*, and *Δrna* vs. *Δrna*+pKT-CNP-Inact; and for: *S*. Typhimurium WT vs. WT+pKT-CNP, WT vs WT+pKT-CNP-Inact, *Δrna* vs. *Δrna*+pKT-CNP, and *Δrna* vs. *Δrna*+pKT-CNP-Inact, (*p*-values <0.05).

REFERENCES

1. Baba T, Ara, T., Hasegawa, M., Takai, Y., Okumura, Y., Baba, M., Datsenko, K. A., Tomita, M., Wanner, B. L., and Mori, H. 2006. Construction of *Escherichia coli* K-12 in-frame, single-gene knockout mutants: the Keio collection. Mol Syst Biol 2.
2. Kurasz JE, Hartman, C. E., Samuels, D. J., Mohanty, B. K., Deleveaux, A., Mrázek, J., and Karls, A. C. 2018. Genotoxic, metabolic, and oxidative stresses regulate the RNA repair operon of *Salmonella enterica* serovar Typhimurium. J Bacteriol 200:e00476-18.
3. Fontaine BM, Martin, K. S., Garcia-Rodriguez, J. M., Jung, C., Briggs, L., Southwell, J. E., Jia, X., and Weinert, E. E. 2018. RNase I regulates *Escherichia coli* 2',3'-cyclic nucleotide monophosphate levels and biofilm formation. Biochem J 475:1491-1506.
